# Rational steering of insulin binding specificity by intra-chain chemical crosslinking

**DOI:** 10.1038/srep19431

**Published:** 2016-01-21

**Authors:** Jitka Viková, Michaela Collinsová, Emília Kletvíková, Miloš Buděšínský, Vojtěch Kaplan, Lenka Žáková, Václav Veverka, Rozálie Hexnerová, Roberto J. Tarazona Aviñó, Jana Straková, Irena Selicharová, Václav Vaněk, Daniel W. Wright, Christopher J. Watson, Johan P. Turkenburg, Andrzej M. Brzozowski, Jiří Jiráček

**Affiliations:** 1 Institute of Organic Chemistry and Biochemistry, the Czech Academy of Sciences, v.v.i., Flemingovo n. 2, 166 10 Praha 6, Czech Republic; 2 York Structural Biology Laboratory, Department of Chemistry, University of York, Heslington, York YO10 5DD, United Kingdom

## Abstract

Insulin is a key hormone of human metabolism with major therapeutic importance for both types of diabetes. New insulin analogues with more physiological profiles and better glycemic control are needed, especially analogues that preferentially bind to the metabolic B-isoform of insulin receptor (IR-B). Here, we aimed to stabilize and modulate the receptor-compatible conformation of insulin by covalent intra-chain crosslinking within its B22–B30 segment, using the Cu^I^-catalyzed Huisgen 1,3-dipolar cycloaddition reaction of azides and alkynes. This approach resulted in 14 new, systematically crosslinked insulin analogues whose structures and functions were extensively characterized and correlated. One of the analogues, containing a B26–B29 triazole bridge, was highly active in binding to both IR isoforms, with a significant preference for IR-B. Our results demonstrate the potential of chemistry-driven modulation of insulin function, also shedding new light on the functional importance of hormone’s B-chain C-terminus for its IR-B specificity.

Insulin is a small protein hormone composed of chain A (21 residues) and chain B (30 residues), and it exerts its biological effects by binding to the ~340 kDa tyrosine-kinase type insulin receptor (IR). Insulin plays a central role in mammalian biology^1^, and has a widespread impact on blood glucose levels, lipid and protein metabolism, cell growth/differentiation, and life span^2^. The IR ectodomain comprises two α-subunits and two β-subunits linked by S-S bonds. The α-subunits assure hormone binding, whereas β-subunits transduce the signal to the cytoplasmic tyrosine-kinases of the IR^3^,^4^. Insulin signaling pathways are complex due to the existence of two IR isoforms: IR-A, and the alternatively spliced IR-B, which has 12 additional amino acids at the C-terminus of the α-subunit^5^, within the so-called αCT-segment. The IR-isoform expression pattern is tissue specific^6^,^7^,^8^, with IR-B being most abundant in the main insulin glycemic-response tissues (e.g., IR-B accounts for 90–95% of all IR in liver), but being also present in muscles and adipose tissue. The IR-A isoform dominates in brain, fetal and cancer tissues, and is also a relatively good receptor for the growth factors IGF-2 and IGF-1. These characteristics make IR-B the predominantly ‘metabolic’ type of the IR, whereas IR-A can act in other, more mitogenic, signaling pathways.

Defects in insulin bio-availability, or impaired IR signaling, may result in different pathological conditions, such as diabetes (type 1 – insulin dependent; type 2 – insulin independent)^9^,^10^, cancers^11^ and Alzheimer’s disease^12^.

Extensive structural and functional characterization of insulin allowed rational optimization of some of its pharmacodynamic properties. It has focused on two, rather contrasting tasks: (i) the delivery of fast-acting insulins (with a highly monomeric character or with rapid dissociation from the hexameric form prevalent in insulin storage), and (ii) the development of long-acting insulins. The second task tries to minimize the frequency of exogenous delivery of the hormone to diabetics through restoration and mimicking of the physiological profile of insulin-dependent glycemic control. Under physiological conditions, approximately half of the insulin secreted from the pancreas to the portal vein is used by the liver-abundant IR-B, resulting in a predominantly hepatic to peripheral action of this hormone. In contrast, exogenously delivered insulin interacts primarily with the homologous IR-A:IR-B distribution in the peripheral tissues, prior to inducing its normal liver response^13^. Because the excessive exposure of nonhepatic tissues to insulin in diabetics can lead to a plethora of significant side effects, the development of novel insulins with high IR-B and low IR-A specificities is highly desirable.

Progress in rational mimicking of the physiological profile and action of insulin has been hampered by lack of 3D structures of its complex with IR. However, the long-awaited structural insight into insulin:IR initial binding events has recently been attained^14^. Surprisingly, the structures of insulin in complexes with some IR constructs showed that the main interaction of the hormone with the N-terminal L1 domain of the IR α-subunit (the main hormone binding site) is not direct, but is mediated extensively by the αCT-segment. Remarkably, the αCT-segment moves in this process across the whole ectodomain, and is anchored on the L1-surface of the neighboring α-subunit. Importantly, these first insulin-IR complex structures also shed light on the ‘active’ conformation of insulin B22–B30 chain^15^, which has to undergo a conformational change on the receptor. Interestingly, some of our parallel efforts to decipher the ‘active’ B22–B30-fold by extensive chemical modifications yielded highly active insulin analogues^16^. The main common signature of these analogues was the so-called B26-turn (a particular bend of the B25–B30 chain), combined with the structural conservation of the invariant and important PheB24 site^17^,^18^. The overall conformational features of this structural convergence were supported by the structures of the insulin:IR complexes. Moreover, we also showed that the IR-complex-compatible B26-turn-like conformation of the B25–B30 insulin chain can be generated by a single natural mutation at the B26 site (e.g., Tyr to Asn)^19^, highlighting further the importance of the B26 region for an effective insulin-IR interaction (Fig. 1). This work also indicates the potential of protein chemistry as an important tool in insulin studies that can provide an independent and reliable insight into insulin structure and function.

Experience with the structural and functional manipulation of insulin, underpinned by the progress in insulin:IR research, encouraged us to probe an induction of the active structure of this hormone by the incorporation of a covalent crosslink within its B22–B30 chain, which would result in a stable B26-turn-like conformation. However, the aims of this research evolved along the promising progress of this research: the initial goal of capturing a ‘mechanistic snapshot’ of insulin conformation was transformed into the aim of design and delivery of IR isoform-specific analogues.

To achieve the stabilization of a specific conformation of the hormone, we developed the strategy of inserting a covalent bridge between specific sites at the B24–B30 C-terminus of the insulin B-chain, i.e., an intramolecular side-chain:side-chain crosslinking. The Cu^I^-catalyzed Huisgen 1,3-dipolar cycloaddition reaction of azides and alkynes^20^,^21^ was employed in this process. This biorthogonal reaction, also called ‘click chemistry’, leads to the formation of 1,4-disubstituted 1,2,3-triazoles. This reaction has become widely used^22^ in organic, medicinal and, especially, peptide chemistry because 1,2,3-triazoles present a motif with structural and electronic characteristics similar to those of the peptide bond. Moreover, 1,2,3-triazoles have found applications in peptide cyclizations, induction of some secondary structures in peptides, especially β-turns^23^,^24^, β-hairpins^25^, helical structures^26^,^27^,^28^ or mimics of disulfide bonds^29^. This approach was recently used to replace the A7-B7 inter-chain disulfide bond in insulin, but it yielded misfolded and inactive protein^30^. A succesfull incorporation of a triazole crosslink into the full protein structure (on an analytical scale) was reported by Neumann *et al.*^31^, who encoded precursor amino acids into the sequence of calmodulin, with the subsequent *in vitro* formation of the crosslink. Here, we selected Cu^I^-catalyzed cycloaddition to bridge specific sites within the insulin B-chain B24–B30 C-terminus (intra-chain crosslinking), using specific amino acids with azido and alkynyl side chain groups as precursors. To our knowledge, the research reported here is a unique example of the use of triazole-forming click chemistry within the same native protein main-chain to successfully steer its structure and function.

This work yielded 14 novel, systematically crosslinked insulin analogues, which structures and functions were extensively characterized. Most interestingly, one of the analogues, insulin with a B26–B29 triazole-bridge, was highly active in binding to both isoforms of IR, with a significant preference for IR-B. Our results indicate the potential of chemistry-driven modulation of insulin function, and also shed light on the functional importance of the B-chain C-terminus of the hormone for its IR-B specificity.

## Results

### Synthesis of the analogues

Insulin analogues **1**–**14** were prepared according to the previously described protocol^16^,^32^ for the enzymatic semisynthesis starting from *des*(B23–B30)octapeptide-insulin (DOI) and cyclic (crosslinked) octapeptides **1b**–**14b**, respectively. In the case of some less soluble cyclic peptides, the semisynthesis was performed according to the slightly modified protocol by Nakagawa and Tager^33^. The solid-phase synthesis of the linear insulin B23–B30 octapeptide precursors **1a**–**14a** proceeded readily and with good yields. These precursors contained azido- and alkynyl-side chain amino acids at different sites as precursors for Cu^I^-catalyzed cycloaddition. Cycloadditions were performed using two methods. Method A was usually completed after 30 min, and the cyclic products were easily purified by HPLC. However, cyclization of the linear B23–B30 precursor **14a** for insulin analogue **14** also yielded a 5-hydroxy-triazole cyclic octapeptide byproduct that was difficult to separate. This issue was overcome by the use of method B by Chen *et al.*^34^; this process uses the TBTA copper ligand and an inert atmosphere in the dark to minimize the formation of hydroxylated triazoles.

**Figure 2:**
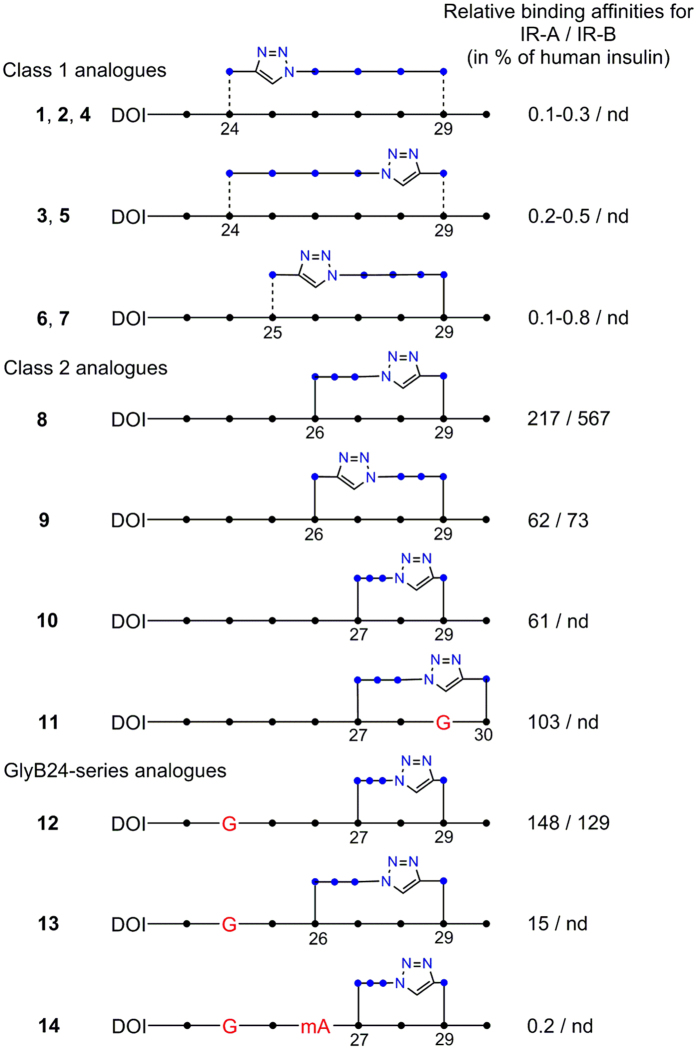
Schematic representation of intra-chain crosslinked octapeptides in the insulin analogues 1–14 produced in this work. Black dots (●) indicate the Cα carbons in the backbone and blue dots (●) indicate CH_2_ atoms in the linkers. Dashed lines indicate alternative chirality. G in red indicates the positions of Gly mutations. mA in red indicates the position of *N*-methylated alanine. The binding affinities (in %) relative to native insulin are shown to the right. Triazole rings are shown as pentagrams with blue nitrogen atoms. nd stands for not determined. DOI is *des*(B23–B30)octapeptide-insulin.

The schematic structures of insulin analogues (**1**–**14**), together with their binding affinities for IR (for the analogue **8** also for the IGF-1R), are summarized in Fig. 2 and in Table 1. The binding curves are shown in Supplementary Fig. S1–S4.

### Design strategies for intra-chain crosslinking of insulin analogues

The original aim and strategy of this research was to probe whether the insulin-active-like conformation (observed in its IR complex and highly active analogues), i.e., bend of the B23–B30 chain around B25–B26 sites, can be mimicked, stabilized and exploited by the incorporation of a crosslink (covalent bridge) within this region of the hormone. The main steric parameters were as follows: (i) the positions of the propargylglycine (Prg) and azidonorleucine/azidonorvaline (Nle(εN_3_)/Nva(δN_3_)) precursors within the B23–B30 chain, (ii) the length of the crosslinked protein main chain, (iii) the chirality or the precursors, and (iv) the position of the triazole ring (i.e., up- or down-stream of the main chain). The chiralities and side-chains related restraints of the B24 and B29 sites were also altered, or liberated, by Gly substitutions in some analogues.

These strategies resulted in 14 analogues that may be grouped into two classes that reflect certain correlations between their affinities and the type of click-crosslinking (Fig. 2). All class 1 analogues (**1**–**7**) have triazole-containing linkers with eight backbone atoms, while all class 2 analogues (**8**–**14**) have triazole-containing linkers with 7 backbone atoms.

#### Class 1: i + 5/i + 4 crosslinked non-active analogues

The long (i + 5) separation between clicking groups was first chosen to assure a wide and gentle sweep of the main chain conformation, i.e., to minimize its structural strain, and to allow the B23–B30 chain to attain a conformation similar to the B26-like turn, or to the bend of the hormone observed in the insulin:IR complexes. However, the IR-A affinities of all of B24–B29 crosslinked analogues **1–5** were negligible (<1%) (Table 1), regardless of the *R*/*S* permutations of the Cα chirality of the crosslinking precursors (propargylglycine and azidonorleucine).

Subsequently, the span of the crosslinked main chain was shortened to i + 4, and the anchor site of the resulting triazole ring was moved from the B24 to the B25 site. However, as in the i + 5 case, analogues **6** and **7** were inactive.

#### Class 2: i + 3/i + 2 crosslinked active analogues

Subsequently, the impact of shorter i + 3 cyclizations between the B26–B29 sites was investigated. The anchor site for the first (sequence-wise) click precursor site was moved further to position B26, and the azidonorleucine click precursor was substituted here by a shorter azidonorvaline acid to facilitate tighter crosslinking. Analogues **8** and **9** that were created during this step differed in the reversed positions of the propargyl and azido precursors; this change was implemented to probe the effect of the position of the triazole ring within a particular analogue on its structure and function. This approach yielded the highly IR-A potent (208 or 226% in two independent measurements) analogue **8**. The move of the triazole position to a location near the B26 site in analogue **9** lowered its IR-A affinity to ~62%.

Because the B24–B29 to B26–B29 shift of the crosslink bridge yielded the high IR-A affinity analogue **8**, possible larger gains in receptor affinity upon moving the crosslink further towards the C-terminus of the B-chain were expected and investigated by the B27–B29 (i + 2) cyclization of insulin. However, this shortening of the crosslinked sequence lowered the affinity of the resulting analogue **10** to 61%.

Analogues **8–10** indicated possible superiority of the i + 3 over i + 2 cyclization. This was examined in the next analogue, **11**, in which the azido precursor at B27 was clicked with propargyl at B30. The side chain-free GlyB29 was used here instead of the wild-type LysB29 to avoid possible steric interference in the cyclization reaction. The full insulin IR-A affinity (103%) was regained in this analogue.

The class 2 crosslinked insulins also contain a sub-group of analogues with an achiral Gly at the B24 site (GlyB24-series). This substitution resulted from an accidental PheB24Gly replacement during one of the preparations of the octapeptide precursor for analogue **10**. Surprisingly, this error generated a 148% IR-A potent analogue **12**, with more than double the affinity compared to its ‘correct’ PheB24 homologue **10**. This result prompted the incorporation of GlyB24 into the most active insulin in this series, analogue **8**, but the resulting analogue **13** showed a potency of only 15%.

The last insulin of this series, analogue **14**, was an attempt to enhance further the affinity of analogue **12,** by the addition of the methylation of the B26 NH-main chain atom previously (and successfully) exploited by us. This modification in the N-MeAlaB26 analogue (truncated also and amidated at the B26 site) yielded a highly active (465%) form of the hormone^16^. However, this more extensive combination of the main- and side-chain modifications together with the B27–B29 crosslink and the GlyB24 mutation was not additive, and practically abolished the affinity of analogue **14**.

### IR-B specificity and IGF-1R binding of crosslinked insulin analogues

The analogues with significant IR-A affinities were selected and tested further for their binding to the IR-B isoform and to the IGF-1R receptor. Most interestingly, the IR-A highly active (~217%) analogue **8** also showed a potency of more than five times (~567%, i.e. 515 or 620% in two independent measurements) for the IR-B isoform. The swap of the crosslink precursor sites in **8** lowered the IR-B/IR-A affinities of the resulting analogue **9** to 73% and ~62%, respectively, indicating the sensitivity of the IR-B specificity to the position of the triazole group, and, likely, to the particular conformation of this B26–B29 crosslink. Moreover, the GlyB24-containing analogue **12** with 148% IR-A affinity was also equipotent against IR-B (129%).

The significance of the desirable IR-A/B affinities of analogue **8** was amplified further by its approximately four times lower potency towards IGF-1R, making this insulin and its chemical scaffold a promising molecular platform for future clinically-oriented studies of this hormone.

### Structure-function correlations of crosslinked insulin analogues

**Figure 3:**
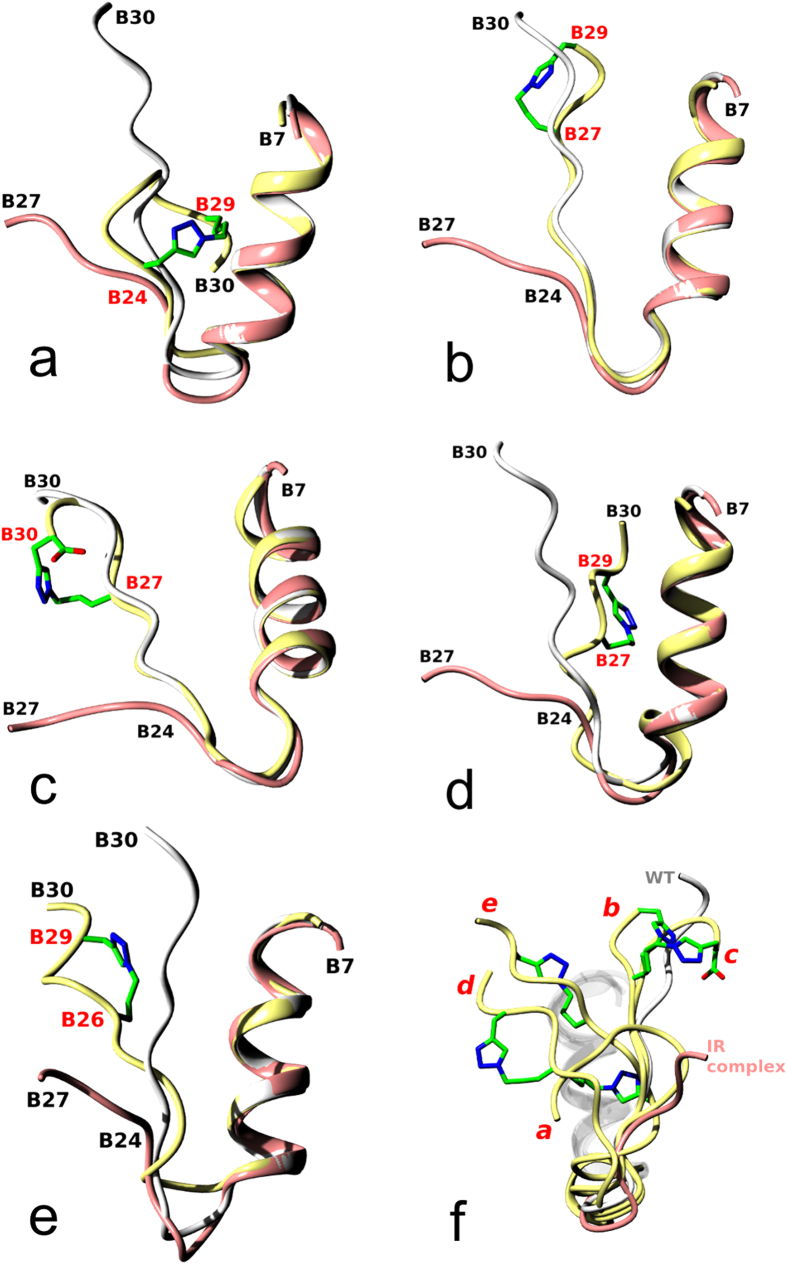
Comparison of range of conformations of the B-chain observed in the structures of the wild-type human insulin (white), insulin in the IR complex (coral), and in the analogues discussed in this work (yellow): (**a**) **2**, (**b**) **10**, (**c**) **11**, (**d**) **12**, and (**e**) **8**. The carbon atoms of the crosslinks are in green, and the nitrogens in blue; crosslinked sites are labeled in red; in (**c**), the C-terminal carboxyl group (part of one crosslink precursor) is also given. The wide spectrum of crosslink-generated conformations of insulins is shown in (**f** ) by superposition of the analogues given in (**a–e**). The red letters at the analogues correspond to the structures in panels (**a–e**); wild-type (WT in white, pdb 1 mso) and IR-complexed insulin (coral, pdb 4 oga) are also given with all B7–B19-helices shown in white.

**Figure 4:**
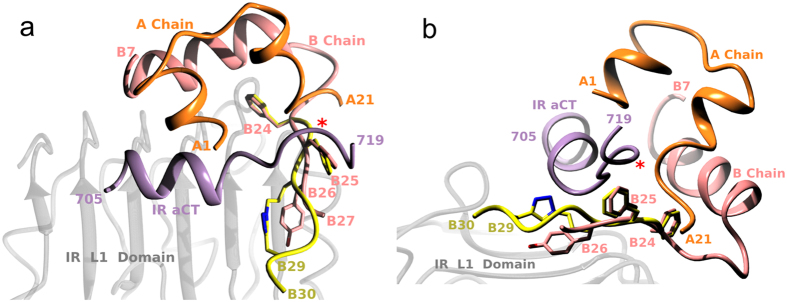
Two views of a putative fold of the B24–B30 crosslink-containing chain of the IR-B-specific analogue 8 modeled on the insulin:IR complex structure, assuming an invariant conformation of PheB24. (**a**) View from the top of the IR L1 domain and (**b**) from the side (edge) of the IR L1 domain. Insulin A and B chains are in gold and coral, respectively, and the αCT IR-A segment in violet. The B24–B30 segment of **8** is in yellow with triazole nitrogens in blue. Red asterisk indicates the insertion site (716) of the exon 11-coded additional 12 amino acids of the αCT segment in the IR-B isoform.

The evidence for the structural impact of the crosslinking in the analogues reported here comes from three crystal (analogues **2**, **10**, **11**) and two NMR (analogues **8**, **12**) structures. Because the B24–B30 chain of insulin undergoes a large conformational change upon IR binding, the structures were also superimposed on the ‘active’ form of the wild-type insulin in its IR complex to provide some general structural context^15^ (Fig. 3). However, to obtain a more realistic assessment of the structural impact of the crosslink on the insulin-embracing IR environment, only the B24–B30 chain of each analogue was superimposed on the receptor-complexed hormone. The conformation of the insulin PheB24 main and side chains in the IR complex (structural and functional ‘pivot’ of the IR-bound hormone) was the main overlap target (Fig. 4).

### Analogue 2 (cyclo[G^23^-Prg-FYTP-Nle(εN_3_)-T^30^]-insulin)

The B24–B29 linker spans a long stretch of the B-chain, strongly affecting the local and global conformation of this inactive analogue (Fig. 3a). First, the linker significantly increases the mobility and disorder of this part of insulin, and it is well defined in only one of four independent hormone molecules in the crystal, with one B-chain untraceable after CysB19. Although the triazole part of the linker (in the most ordered molecule) occupies roughly the PheB24 site of the native insulin, the B23–B30 main chain is very distorted (e.g. 3.8 Å difference at the B25 Cα atoms from the native hormone). However, it seems that not the increased dynamics of the B20–B30 chain but a peculiar conformation of the large B24–B29 ‘macrocycle’ of this analogue abolishes its IR-A affinity. Any modeling of analogue **2** (or its B24–B30 part only) on the insulin:IR complex leads to large clashes of the B24–B29 loop with the L1 surface of the receptor, or its αCT segment. This extensive steric hindrance occurs despite the general, and reasonable, match between the triazole moiety and the PheB24 site on the L1 domain. However, it is also possible that the polar character of the electron-rich triazole ring is incompatible with the PheB24 hydrophobic binding pocket (Asn15, Leu37, Phe39 and Phe714) on the IR. Therefore, the lack of any IR-A affinity of analogue **2** likely can result from both the disruption of the B24 site (on insulin and IR), and an unfavorable conformation of the large B24–B29 macrocycle on the αCT-L1-domain.

### Analogue 10 (cyclo[G^23^FFY-Nva(δN_3_)-P-Prg-T^30^]-insulin)

The B24–B29 to B27–B29 shift of the crosslinking bridge almost restored the IR-A affinity (61%) of this analogue. Interestingly, the B27–B29 linker does not significantly affect the overall or local (e.g., B20–B26) insulin structures (Fig. 3b); the linker allows even the formation of typical insulin dimers with usual dimer-interface hydrogen bond patterns. Any structural variability observed here is characteristic for all other known insulin oligomers. However, in contrast to **2**, the B24–B30 chain of analogue **10**, when modeled on the B24 site of IR-bound insulin (not shown), can, somehow, be accommodated on the L1 surface, and its further reconfiguration will diminish clashes with the αCT-segment. Therefore, the shortening of the crosslinked sequence and its shift towards the very end of the B-chain in analogue **10** alleviates the conformational stress observed in **2**, allowing likely a more effective contribution of the B24–B26 part of the B-chain towards its interaction with IR-A.

### Analogue 11 (cyclo[G^23^FFY-Nva(δN_3_)-PG-Prg^30^]-insulin)

The full IR-A affinity (103%) was restored in analogue **11** by expansion of the crosslink from position B29 to the B30 site. The LysB29→Gly mutation removed also possible side-chain interference at this site. In contrast to analogue **10**, the crosslinked-loop is not in the plane of the B-chain; instead, it bulges sideways to accommodate the longer linker (Fig. 3c). However, as in **10**, the cyclization within the end of the B-chain did not interfere with the dimeric properties of **11**, allowing this analogue to maintain a regular dimer interface, and to form a typical R_6_ hexamer. Moreover, simple modeling of the B24–B30 segment of **11** on the insulin in the IR-A complex leads to an even more optimum conformation of the B27–B30 crosslinked-loop of this analogue in relation to the L1-domain and the αCT-segment of the receptor. In this model (not shown), the C-terminal part of the B-chain in **11** passes under the αCT-segment and folds back to embrace that segment on the other side of the αCT helix.

### Analogue 12 (cyclo[G^23^GFY-Nva(δN_3_)-P-Prg-T^30^]-insulin)

The NMR structure of this analogue revealed the down-register shift of the B25–B30 insulin chain, observed in previously described GlyB24-containing analogues. All converged structures of **12,** measured at acidic pH, are shown in Supplementary Fig. S5, and a representative structure (at pH 1.9) is shown in Fig. 3d. They confirm the observations that the emptying of the PheB24 cavity in insulin by PheB24Gly mutation is structurally preserved by the pull down of PheB25 into the B24 site^18^,^35^. However, the mimicking of the PheB24 site by PheB25 is less perfect in analogue **12** than in other similar (i.e., PheB24-‘empty pocket’) analogues, and the B25–B30 chain departs sharply from the core of the molecule at ca. 90° angle. The modeling of the standalone B24–B30 chain on the insulin-IR complex (not shown) results in the arrangements similar to those found in analogues **10** and **11**. Analogue **12** confirms also that high IR-affinity does not have to be compromised even in very substantially engineered insulins, if the ring in the B24 site is hydrophobic.

### Analogue 8 (cyclo[G^23^FF-Nva(δN_3_)-TP-Prg-T^30^]-insulin)

The B26–B29 crosslink with the triazole moiety close to the B29 site yielded analogue **8** with 208–226% affinity for IR-A, and, more importantly, 515–620% affinity for the IR-B isoform. As this analogue resisted crystallization, NMR structures were determined in both acidic (pH 1.9) and alkaline (pH 8.0) environments. Supplementary Fig. S6 shows all converged NMR structures of **8** at pH 1.9 and 8.0. The structures are similar and homologous to native insulin up to the PheB24 site, with its phenyl moiety still occupying the PheB24 pocket. However, the remaining B-chain is fully detached from the core of the hormone from the B25 site (exemplified, for example, by the ~16 Å separation between the B25 Cα atoms of the native hormone and analogue **8**). The representative NMR structure of **8** at pH 8.0 is shown in Fig. 3e. Although the conformation of the B26–B30 chain in **8** does not exactly resemble the B26-turn- or AsnB26-bend-like ‘active’ folds, it confirms the trend and scale of the detachment of the B-chain observed in these ‘active’ analogues. The overall conformation of B26-turn-, AsnB26-analogues and analogue **8** B24–B30 chains can be matched by an ~180° rotation about the psi angle of PheB25 in **8** (not shown). Interestingly, the i + 3 crosslink does not cause any bulge of the B-chain (as observed in the i + 3 crosslink of analogue **11**); instead, this crosslink is well accommodated in **8** within the plane determined by the bend of the B-chain.

The structural origins of the high IR affinities in analogue **8** may result, at least partly, from a peculiar and unique conformation of its B24–B30 segment. Firstly, the modeling of only this part of **8** on its equivalent in the IR-complexed insulin (Fig. 4) gives the best fit of all analogues studied here; both chains show a remarkably similar fold and direction if they are matched at the B24 site. Secondly, the rotamer of PheB24, which changes significantly upon insulin binding to IR^15^, is most similar between analogue **8** and IR-complexed insulin.

## Discussion

The research reported here provides the first evidence that the intra-chain Cu^I^-catalyzed cycloaddition (‘click’) chemistry can be used for a rational steering of protein functionality. A series of systematic chemical crosslinks spanning different sites of insulin’s main-chain led to the development of analogue **8** with high IR affinity, and, more importantly, with a significant selectivity for the IR-B isoform that is responsible for the insulin main hepatic response.

The structure-function correlations and trends observed in these analogues indicate that the main part of the B-chain responsible for an efficient crosslinking covers the B26–B29 or B27–B29/B30 region of insulin. Both i + 3/i + 2 main-chain separations of click precursors are also efficient in this process, with the i + 3 B26–B29 crosslink generating both high affinity and significant IR-B receptor selectivity. Furthermore, the particular position of the triazole moiety within the crosslink seems to play an important role in this process as well. All active analogues showed a preference for distal positioning of the triazole in the linker, i.e. with the triazole group closer to the C-terminus of the B-chain. For example, the swap of the triazole anchoring from the B29 site to the B26 site in analogues **8** and **9**, respectively, resulted in a decrease of more than three times in IR-A affinity and a more than seven times lower IR-B affinity. This indicates that inserting triazole-containing linkers into the insulin B-chain not only affects the 3D fold but also creates new sub-structures that are involved in the direct interactions with the IR.

However, despite some clear structural and functional trends, the actual structural origins of higher affinity of any of these analogues are not unambiguous. This is not surprising as the large-scale of the conformational activation of insulin B24–B30 chain upon binding to IR prevents meaningful, direct superposition of the analogues on the native insulin in the IR-A complex. However, modeling the B24–B30 segments with the IR-complexed insulin provides valuable insight into the role of the crosslink in generation of different affinities. For example, a better accommodation of the B24–B30 chain between the L1-surface and αCT-segment correlates well with the higher affinity of an analogue, although further, small structural tuning of the modelled B24–B30 chains is certainly required for their more effective tethering into the IR. The role of a particular rotamer of PheB24 (observed in the insulin:IR complex)^15^ may be crucial in this process as well. Nevertheless, the crosslink-induced macrocycles in the B26–B30 regions of the analogues provide a novel, adaptable, but ultimately quite rigid (i.e., triazole group) scaffold for the putative interactions with both the L1-surface and αCT-segment.

The structural reasons for the remarkably high (~567%) affinity of analogue **8** for IR-B require further studies, and are currently unclear; there are no obvious, dominating putative interactions between the modeled B25–B30 chain of analogue **8** and the αCT-segment. However, the site of a 12-amino acid insertion (exon11) and hence the extension of the αCT-polypeptide in the IR-B isoform (Pro716) lies just above the PheB25-TyrB26 region of insulin. Therefore, it cannot be excluded that the longer αCT-B-segment does not simply follow the conformation of αCT-A (i.e., departing from the L1 domain). Instead, this segment might adopt a different conformation, which, for example, would be more engaged with the (possibly rearranged) B26–B29 crosslinked loop of analogue **8**. These new (but still unobserved) IR-B:**8** specific contacts may involve both the triazole ring and insulin B27–B30 main/side-chains of this structurally stable and new ‘macrocycle’ within this hormone. The importance of the position/orientation of the triazole ring for the interaction with IR is also evident from the lower binding affinities of **9** (a homologue of **8**), in which the triazole was placed closer to the B26 site. Interestingly, it was suggested that the almost four-times IR-B specificity of the triple HisA8, HisB25, and GluB27 mutant of insulin (15% for IR-A, 57% for IR-B) can originate from the putative intra-molecular hydrogen bond between the side chains of the mutated B25–B27 sites^36^. Therefore, it cannot be excluded that the crosslink in **8** may reflect similar, but firmer, covalent intra-molecular stabilizing interactions.

In summary, we show here that the combined application of organic and protein chemistry to the B26–B30 ‘IR-irrelevant’ part of the hormone modulates its functionality, and transforms this region into a structural element that confers high affinity and specificity to insulin. The incorporation of specific triazole crosslinks into the C-terminal part of the B-chain of insulin can change the conformation of this insulin segment, initiating its detachment from the core of the insulin molecule. Importantly, this modification provides new, chemical and artificial protein sub-structures that interact with IR and modulate insulin affinity. Therefore, the triazole-containing crosslinks (or other suitable chemical moieties) in the insulin B-chain C-terminus could become promising synthetic platforms for the rational design of receptor-selective insulins, especially when the engineered part of insulin can be easily attached to the rest of the hormone by enzymatic semisynthesis. The incorporation of specifically positioned crosslinks provides a new path for more extensive applications of organic chemistry in engineering of the key elements of insulin pharmacophore, and ultimately, an excting possibility for isoform-selective analogues.

## Methods

### Solid-phase synthesis

Synthetic linear octapeptides (**1a**–**14a**), which were derivatives of the C-terminal octapeptide of the B-chain of human insulin, were prepared by manual solid-phase synthesis on a 2-chloro-chlorotrityl resin using HBTU/DIPEA reagents and Fmoc-protected amino acids. Fmoc-L-propargylglycine, Fmoc-D-propargylglycine, Fmoc-L-N^ε^-azidonorleucine, Fmoc-D-N^ε^-azidonorleucine or Fmoc-L-N^δ^-azidonorvaline was incorporated at different positions in the peptides.

### Cycloaddition reactions

The cyclic octapeptides **1b**–**14b** were prepared from the linear precursors **1a**–**14a**, respectively, by Cu^I^-catalyzed cyclization using two different methods, A and B, as follows.

### Method A

The click reactions with octapeptides **1a**–**13a** were performed according to the protocol previously published by Isaad *et al.*^37^. Briefly, the linear octapeptide (~50 mg, 0.08 mM) was dissolved in a water:*tert*-butanol mixture (2:1, v/v). Then, ascorbic acid (14 eq) and CuSO_4_.5 H_2_O (14 eq) were added to a minimum volume of the water:*tert*-butanol mixture. The reaction mixture was briefly stirred, and then the reaction proceeded without stirring at RT. The reaction was monitored by HPLC. After the completion of the reaction, the reaction mixture was concentrated in vacuum to remove *tert*-butanol, and the products were isolated from the residual water phase and salts using solid-phase extraction on C18-cartridges (Macherey-Nagel) and purified using HPLC.

### Method B

The cyclization click reaction with octapeptide **14a** was performed differently^34^. The linear octapeptide **14a** (~50 mg, 0.08 mM) was dissolved in a degassed mixture of water and *tert*-butanol (2:1, v/v). Then, ascorbic acid (10 eq), CuSO_4_.5 H_2_O (5 eq) and TBTA (5 eq) were premixed and added to a minimum volume of the degassed water/*tert*-butanol mixture. The reaction mixture was briefly stirred, and then the reaction proceeded without stirring at RT in the dark under an inert atmosphere. The next steps were the same as described for method A.

### Enzymatic semisynthesis

The insulin analogues **1**–**14** were prepared according to the previously described protocol^16^,^32^ for the enzymatic semisynthesis starting from *des*(B23–B30)octapeptide-insulin (DOI) and cyclic octapeptides **1b**–**14b**, respectively. In the case of some of the less soluble cyclic peptides, the semisynthesis was performed according to the slightly different protocol published by Nakagawa and Tager^33^.

### Peptide characterization

The precursor peptides (**1a**, **3a**–**9a**, **11a**–**14a** and **1b**–**14b**) and the resulting insulin analogues **1**–**14** were purified by HPLC. The purity of the products was higher than 95% in all cases. Crude linear peptides **2a** and **10a** were used for the synthesis of their respective cyclic counterparts **2b** and **10b** without HPLC purification. The identity of all precursor octapeptides and resulting analogues was confirmed by MS (LTQ Orbitrap XL, Thermo Fisher Scientific, Waltham, MA, USA).

^1^H and ^13^C NMR spectra were recorded for octapeptides **1a**, **6a** and **1b**–**14b**. The NMR spectra were measured on a Bruker AVANCE-600 instrument (^1^H at 600.13 MHz and ^13^C at 150.9 MHz) with a cryoprobe in H_2_O:D_2_O (9:1) at 25 °C. A trace amount of dioxane was added as an internal standard, and spectra were referenced using δH (dioxane) = 3.75 ppm and δC (dioxane) = 69.3 ppm. Structural assignment of proton and carbon signals was achieved by combining 1D–1H and 1D-APT-13C-spectra with homonuclear 2D-H,H-COSY, 2D-H,H-TOCSY, 2D-H,H-ROESY and heteronuclear 2D-H,C-HSQC and 2D-H,C-HMBC spectra. The temperature coefficients of the amide NH protons were obtained from additional ^1^H NMR measurements at 5°, 15° and 35 °C.

Details about the syntheses and analyses of all linear and cyclic octapeptide precursors, the resultant insulin analogues and their chemical structures and analytical data are provided in the Supplementary Information (Supplementary text, Fig. S7–S21 and Tables S1–S7).

### Determination of NMR structures of analogues 8 and 12

All NMR data were acquired at 25 °C from a 0.35 ml sample of 1.5 mmol.l^−1^ analogue **8** or **12** in a 20% d_4_-acetic acid (pH 1.9) or in a 25 mmol.l^−1^ deuterated-Tris buffer (pH 8.0) containing 5% D_2_O/95% H_2_O for analogue **8** only on a 600 MHz Bruker Avance spectrometer equipped with a triple-resonance (^15^N/^13^C/^1^H) cryoprobe. A series of homonuclear spectra were recorded to determine sequence-specific resonance assignments for the analogues. In particular, 2D TOCSY with a 60 ms mixing time, 2D DQF-COSY and 2D NOESY, which was acquired with an NOE mixing time of 200 ms. The resonance assignments were deposited in the BMRB database. Residues involved in forming stable backbone hydrogen bonds were identified by monitoring the rate of backbone amide exchange in the 2D TOCSY spectra of both analogues dissolved in 20% d_4_-acetic acid/80% D_2_O. The family of converged structures for both analogues was initially calculated using Cyana 2.1. A combined automated NOE assignment and structure determination protocol was used to automatically assign the NOE cross-peaks identified in the 2D NOESY spectrum and to produce preliminary structures^38^. Subsequently, five cycles of simulated annealing combined with redundant dihedral angle constraints (Redac)^39^ were used to produce a set of converged structures with no significant restraint violations (distance and van der Waals violations <0.5 Å); these structures were further refined in explicit solvent using the YASARA software with the YASARA force field^40^. The 30 structures with the lowest total energy were selected. The family of obtained structures was analyzed using the Molmol^41^, Protein structure validation software suite (PSVS)^42^ and PyMol. The numbers of observed NOE peaks, additional constraints and structural statistics for the final water-refined sets of structures are shown in Supplementary Tables S8 (analogue 8) and S9 (analogue 12).

### Crystallization, data processing and structure determination

Crystal growth conditions were found by the use of an in-house insulin-oriented crystallization screen. Data collection and refinement statistics are given in Supplementary Table S10. All crystals were grown by the hanging-drop method at 292K, with 1:1 and 1:2 protein:well ratios and 5 mg/ml as the main concentration of the analogues in 20 mM HCl. Crystallization conditions: (*i*) analogue **2**–0.1 M (NH_4_)_2_SO_4_, 1% (v/v) dioxane, pH 3.0; (*ii*) analogue **10**–0.05 M Li_2_SO_4_, pH 3.0; and (*iii*) analogue **11**–0.6 M Na_2_SO_4_, 0.3 M Tris pH 7.5, 0.6 mM Zn(Ac)_2_, 0.06% (w/v) phenol. X-ray diffraction data were collected at the Diamond Light Source, Didcot (Oxfordshire), UK (analogue **2**: station I03 (ADSC detector, λ = 0.9763 Å); analogue **10**: station I02 (PILATUS detector, λ = 0.9795 Å)), and at the ESRF, Grenoble (analogue **11**: ID23-2, MAR CCD detector, λ = 0.8726 Å). All crystals were directly immersed in liquid N_2_ prior to data collection at 100 K. Measured reflection intensities for all data sets were indexed, integrated and scaled using *XDS*^43^,^44^, HKL2000 (ref. 45) and the *CCP*4 suite^46^ implementation of *AIMLESS*. All structures were solved by molecular replacement with *Molrep*^47^ using B1–B6 and B24–B30 truncated native human insulin (pdb code 1mso) as a phasing model. The structures were refined to convergence *via* the maximum-likelihood method using *REFMAC*^48^, with additional manual correction using *Coot*^49^. The validation of the models in Coot indicated preferred/allowed ratios (%) of 98.2/1.8, 92.9/7.1 and 99.2/0.8 (outlier) for analogues **2**, **10** and **11**, respectively. All structural comparisons between insulins and analogues were performed using the LSQ fit option for the B9–B19 helix Cα atoms in Coot. Modeling of the B24–B30 segment of analogue **8** on the insulin:IR complex (pdb code 4oga) was performed using the best fit of the PheB24 atoms in analogue **8** and complexed insulin. All figures were generated using the *CCP*4*mg* program^50^.

### Receptor Binding Studies

#### Human IM-9 lymphocytes (human IR-A isoform)

Receptor binding studies with the insulin receptor in membranes of human IM-9 lymphocytes (containing only human IR-A isoform) were performed and *K*_d_ values determined according to the procedure described recently in detail by Morcavallo *et al.*^51^. Binding data were analyzed using the Excel algorithms specifically developed for the IM-9 cells system in the laboratory of Prof. Pierre De Meyts (A. V. Groth and R. M. Shymko, Hagedorn Research Institute, Denmark, a kind gift of P. De Meyts) using a method of non-linear regression and a one-site fitting program and taking into account potential depletion of free ligand. Each binding curve was determined in duplicate and the final dissociation constant (*K*_d_) of an analogue was calculated from at least three (n ≥ 3) binding curves (*K*_d_ values) determined independently. The dissociation constant of human ^125^I-insulin was set to 0.3 nM.

#### Mouse embryonic fibroblasts (human IR-B isoform)

Receptor binding studies with the insulin receptor in membranes of mouse embryonic fibroblasts derived from IGF-1 receptor knock-out mice and that solely expressed the human IR-B isoform were performed as described in detail previously^19^. Binding data were analyzed and the dissociation constant (*K*_d_) was determined with GraphPad Prism 5 software using a method of non-linear regression and a one-site fitting program and taking into account potential depletion of free ligand. *K*_d_ values of analogues were determined by the same procedure as for IR-A. The dissociation constant of human ^125^I-insulin was set to 0.3 nM.

#### Mouse embryonic fibroblasts (human IGF-1R)

Receptor binding studies with the IGF-1 receptor in membranes of mouse embryonic fibroblasts derived from IGF-1R knock-out mice and transfected with human IGF-1R were performed as described previously^52^. Binding data were analyzed and the dissociation constants determined by the same method as for IR-B. The dissociation constant of human ^125^I-IGF-1 was set to 0.2 nM. Mouse embryonic fibroblasts expressing human IR-B or IGF-1R were a kind gift of Prof. Antonino Belfiore (University of Magna Grecia, Catanzaro, Italy) and Prof. Renato Baserga (Thomas Jefferson University, Philadelphia, PA, USA).

## Additional Information

**Accession codes:** Atomic coordinates and experimental data sets have been deposited in PDB under following accession codes: 5boq—analogue **2**; 5bpo—analogue **10**; 5bqq—analogue **11**, 2n2v (pH 1.9) and 2n2w (pH 8)—analogue **8;** and 2n2x (pH 1.9)—analogue **12**. Assigned chemical shifts for the solution structures were deposited in BMRB under following codes: 25613 (pH 1.9); 25614 (pH 8)—analogue **8**; and 25615 (pH 1.9)—analogue **12**.

**How to cite this article**: Viková, J. *et al.* Rational steering of insulin binding specificity by intra-chain chemical crosslinking. *Sci. Rep.* **6**, 19431; doi: 10.1038/srep19431 (2016).

## Supplementary Material

Supplementary Information

## Figures and Tables

**Figure 1:**
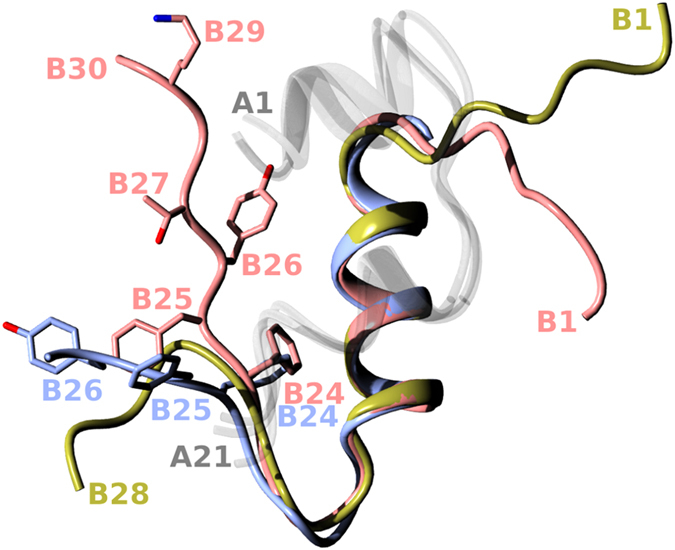
An overlay of human insulin structures that were key templates in this work. All A chains (not relevant here) are in a translucent white; the wild-type B-chain (pdb 1mso) is in coral; the B-chain insulin structure attained in the complex with IR (pdb 4oga) is in blue; and the B-chain of one of the representative B26-turn-containing analogues (AsnB26-insulin, pdb 4 ung) is in gold.

**Table 1:** Crosslinked insulin analogues and their binding affinities.

Protein Position and type of triazole bridge	*K*_d_ ± S.E. [nM] (n) for human IR-A	Relative binding affinity for human IR-A (%)^a^	*K*_d_ ± S.E. [nM] (n) for human IR-B	Relative binding affinity for human IR-B (%)	*K*_d_ ± S.E. [nM] (n) for human IGF-1R	Relative binding affinity for human IGF-1R (%)
Human insulin	0.20-0.43^b^	100c,d,e,f,g,^h^	0.67 and 0.31^i^	100j,^k^	292 ± 31 (3)	0.08 ± 0.01
Human IGF-1	nd^l^	nd	nd	nd	0.24 ± 0.05 (5)	100 ± 21
Cyclo[G^B23^-d-Prg-FYTP-Nle(εN_3_)-T^B30^]-insulin (**1**)	92.0 ± 6.1 (4)	0.33 ± 0.02^c^	nd	nd	nd	nd
Cyclo[G^B23^-Prg-FYTP-Nle(εN_3_)-T^B30^]-insulin (**2**)	226 ± 16 (4)	0.13 ± 0.01^c^	nd	nd	nd	nd
Cyclo[G^B23^-d-Nle(εN_3_)-FYTP-Prg-T^B30^]-insulin (**3**)	59.6 ± 8.2 (4)	0.50 ± 0.07^c^	nd	nd	nd	nd
Cyclo[G^B23^-d-Prg-FYTP-d-Nle(εN_3_)-T^B30^]-insulin (**4**)	126 ± 16 (3)	0.29 ± 0.04^d^	nd	nd	nd	nd
Cyclo[G^B23^-d-Nle(εN_3_)-FYTP-d-Prg-T^B30^]-insulin (**5**)	214 ± 50 (3)	0.17 ± 0.04^d^	nd	nd	nd	nd
Cyclo[G^B23^F-Prg-YTP-Nle(εN_3_)-T^B30^]-insulin (**6**)	32.9 ± 2.6 (3)	0.82 ± 0.06^e^	nd	nd	nd	nd
Cyclo[G^B23^F-d-Prg-YTP-Nle(εN_3_)-T^B30^]-insulin (**7**)	519 ± 104 (3)	0.05 ± 0.01^e^	nd	nd	nd	nd
Cyclo[G^B23^FF-Nva(δN_3_)-TP-Prg-T^B30^]-insulin (**8**)	0.19 ± 0.02 (3) 0.13 ± 0.01 (3)	226 ± 24^f^ 208 ± 16^e^	0.13 ± 0.02 (3) 0.05 ± 0.01 (5)	515 ± 79^j^ 620 ± 124^k^	1327 ± 461 (3)	0.02 ± 0.01
Cyclo[G^B23^FF-Prg-TP-Nva(δN_3_)-T^B30^]-insulin (**9**)	0.56 ± 0.03 (3)	62.5 ± 3.3^g^	0.92 ± 0.12 (4)	73 ± 10	nd	nd
Cyclo[G^B23^FFY-Nva(δN_3_)-P-Prg-T^B30^]-insulin (**10**)	0.33 ± 0.01 (3)	61.0 ± 1.8^h^	nd	nd	nd	nd
Cyclo[G^B23^FFY-Nva(δN_3_)-PG-Prg^B30^]-insulin (**11**)	0.36 ± 0.06 (4)	103 ± 17^d^	nd	nd	nd	nd
Cyclo[G^B23^GFY-Nva(δN_3_)-P-Prg-T^B30^]-insulin (**12**)	0.29 ± 0.06 (3)	148 ± 31^f^	0.52 ± 0.08 (3)	129 ± 20	nd	nd
Cyclo[G^B23^GF-Nva(δN_3_)-TP-Prg-T^B30^]-insulin (**13**)	1.76 ± 0.19 (4)	15.3 ± 1.7^e^	nd	nd	nd	nd
Cyclo[G^B23^GF(*N*Me)A-Nva(δN_3_)-P-Prg-T^B30^]-insulin (**14**)	106 ± 7 (3)	0.19 ± 0.01^h^	nd	nd	nd	nd

The analogues were cyclized at different positions of the C-terminal octapeptide (B23–B30) of the B-chain using specific amino acid precursors: l- or d-propargylglycine (Prg or d-Prg, respectively), l- or d-azidonorleucine (Nle(εN_3_) or d-Nle(εN_3_), respectively) and azidonorvaline (Nva(δN_3_)). Other non-natural substitutions (G for glycine and (*N*Me)A for *N*-methyl-alanine) incorporated into C-terminal octapeptides are also shown. The *K*_d_ values and the relative binding affinities^a^ of human insulin and insulin analogues for isoform A (IR-A) and isoform B (IR-B) of the human insulin receptor or for the IGF-1 receptor (IGF-1R) are also given. Each value represents the mean ± S.E. of (n) independent measurements.

^a^The relative receptor binding affinity (potency) is calculated as *K*_d_ of human insulin/*K*_d_ of analogue) × 100.

^b^Different measurements in the range 0.20-0.43 nM.

^c^Relative to the human insulin *K*_d_ value of 0.30 ± 0.01 (n = 4, 100 ± 3%).

^d^Relative to the human insulin *K*_d_ value of 0.37 ± 0.03 (n = 5, 100 ± 8%).

^e^Relative to the human insulin *K*_d_ value of 0.27 ± 0.04 (n = 5, 100 ± 15%).

^f^Relative to the human insulin *K*_d_ value of 0.43 ± 0.01 (n = 5, 100 ± 2%).

^g^Relative to the human insulin *K*_d_ value of 0.35 ± 0.06 (n = 4, 100 ± 17%).

^h^Relative to the human insulin *K*_d_ value of 0.20 ± 0.02 (n = 5, 100 ± 10%).

^i^Two different measurements.

^j^Relative to the human insulin *K*_d_ value of 0.67 ± 0.08 (n = 4, 100 ± 12%).

^k^Relative to the human insulin *K*_d_ value of 0.31 ± 0.07 (n = 6, 100 ± 23%).

^l^Not determined.
