# Supplementary material for: Rational steering of insulin binding specificity by intra-chain chemical crosslinking
Source: Sci Rep. 2016 Jan 21;6:19431. doi: 10.1038/srep19431 (PMC4726324; doi:10.1038/srep19431)
Supplement: Supplementary Information [file srep19431-s1.pdf]

Supplemental Information

Rational steering of insulin binding specificity by intra-chain chemical crosslinking

Jitka Viková[1], Michaela Collinsová[1], Emília Kletvíková[1], Miloš Buděšínský[1], Vojtěch Kaplan[1], Lenka Žáková[1], Václav Veverka[1], Rozálie Hexnerová[1], Roberto J. Tarazona Aviñó[1], Jana Straková[1], Irena Selicharová[1], Václav Vaněk[1], Daniel W. Wright[2], Christopher J. Watson[2], Johan P. Turkenburg[2], Andrzej M. Brzozowski[2*] & Jiří Jiráček[1*]

[1]Institute of Organic Chemistry and Biochemistry, the Czech Academy of Sciences, v.v.i., Flemingovo n. 2, 166 10 Praha 6, Czech Republic. [2]York Structural Biology Laboratory, Department of Chemistry, University of York, Heslington, York YO10 5DD, England.
Correspondence and request for material should be addressed to J.J. (e-mail: jiracek@uochb.cas.cz) or to A.M.B. (email: marek.brzozowski@york.ac.uk)

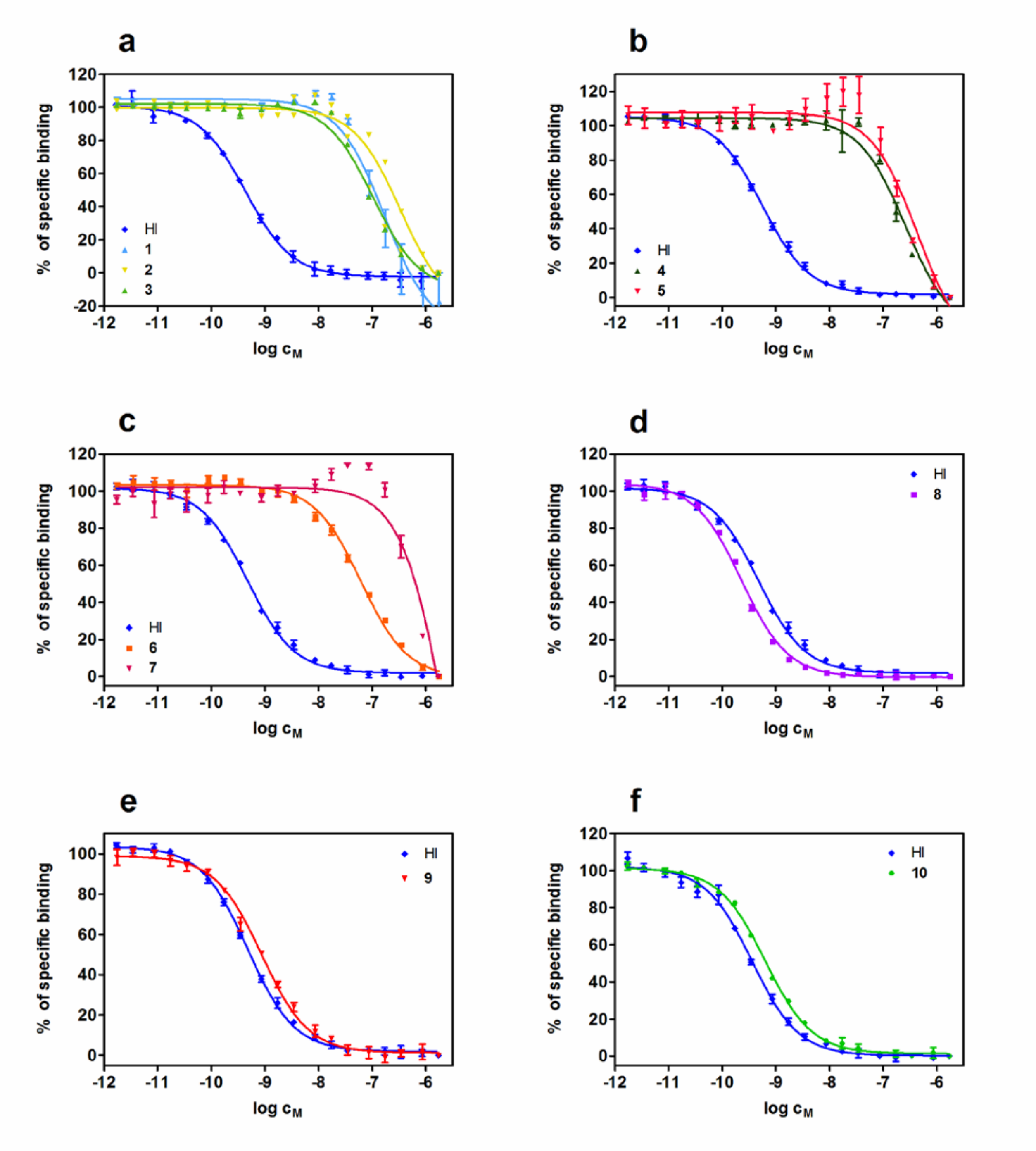

Supplementary Figure S1. Inhibition of binding of human [^{125}I]monoiodotyrosylA14-insulin to IR-A isoform in membranes of human IM-9 lymphocytes by human insulin and insulin analogues. To assure the concision of the SI, only the typical, representative examples of binding curves are shown in Figures S1-S4. In all panels the binding curves of human insulin (HI) are in blue. (a) Analogue **1** (light blue), analogue **2** (yellow) and analogue **3** (green). (b) Analogue **4** (dark

green) and analogue **5** (red). (c) Analogue **6** (orange) and analogue **7** (red). (d) Analogue **8** (violet). (e) Analogue **9** (red). (f) Analogue **10** (green).

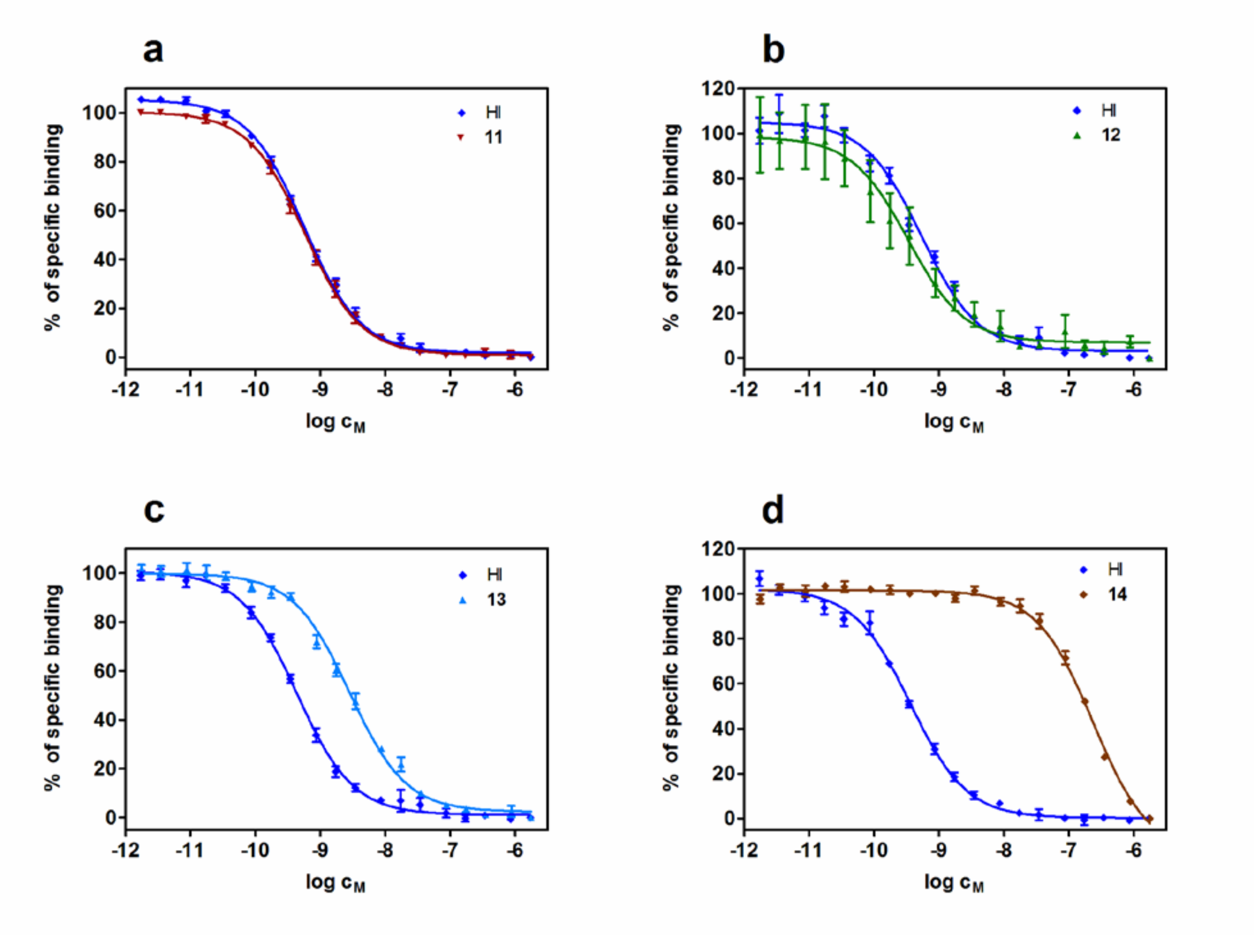

Supplementary Figure S2. Inhibition of binding of human [^{125}I]monoiodotyrosylA14-insulin to IR-A isoform in membranes of human IM-9 lymphocytes by human insulin and insulin analogues. In all panels the binding curves of human insulin (HI) are in blue. (a) Analogue **11** (red). (b) Analogue **12** (green). (c) Analogue **13** (light blue). (d) Analogue **14** (brown).

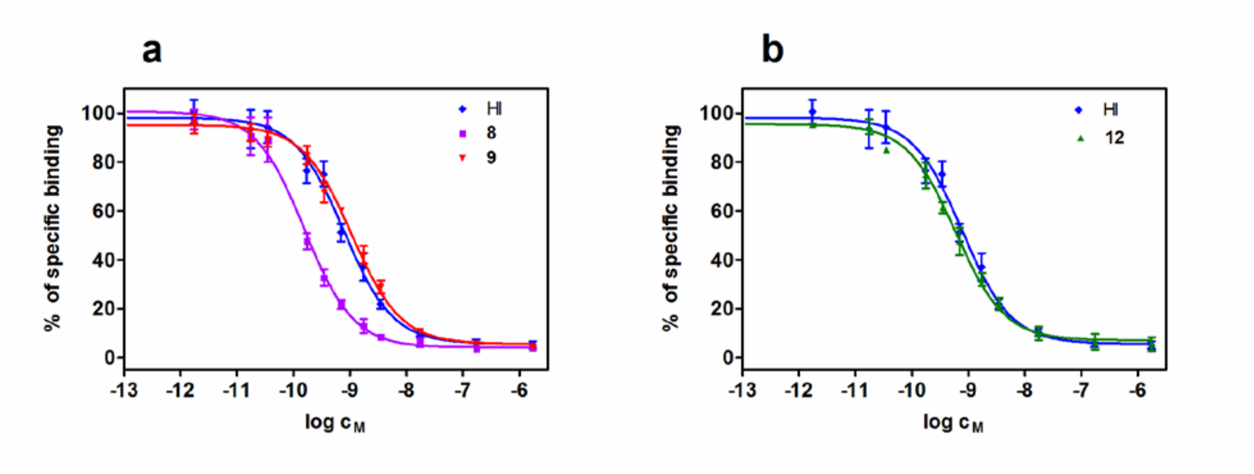

Supplementary Figure S3. Inhibition of binding of human [^{125}I]monoiodotyrosylA14-insulin to IR-B isoform in membranes of mouse embryonic fibroblasts by human insulin and insulin analogues. In all panels the binding curves of human insulin (HI) are in blue. (a) Analogue **8** (violet) and analogue **9** (red). (b) Analogue **12** (green).

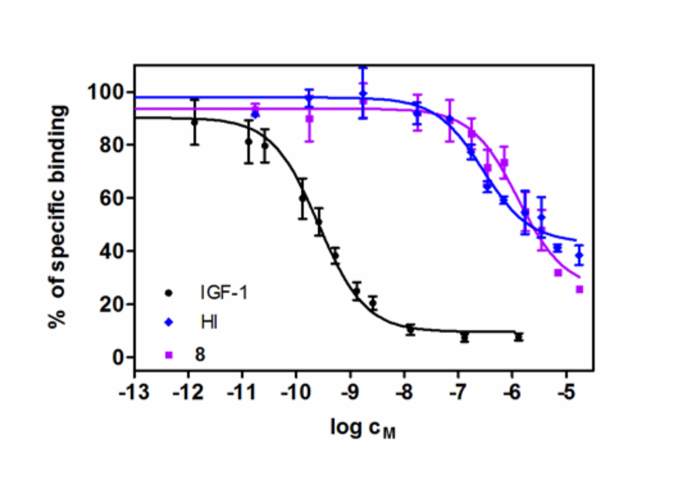

Supplementary Figure S4. Inhibition of binding of human [^{125}I]monoiodotyrosyl-IGF-1 to IGF-1R in membranes of mouse embryonic fibroblasts by human IGF-1, human insulin and insulin analogue 8. Human IGF-1 is in black, human insulin (HI) is in blue and analogue **8** is in violet.

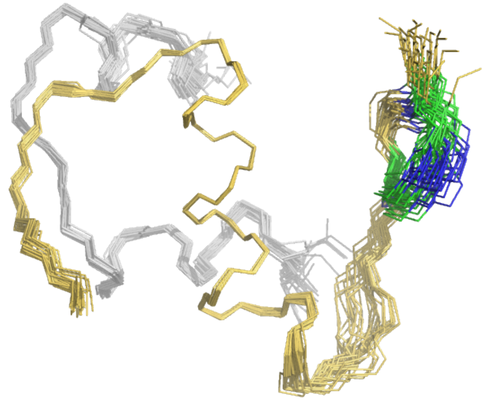

Supplementary Figure S5. Set of converged NMR structures for analogue 12 at pH 1.9. Insulin A chain is shown in gray, B chain is shown in yellow with the triazole cross-link at position B27-29 in green (carbon atoms) and blue (nitrogen atoms).

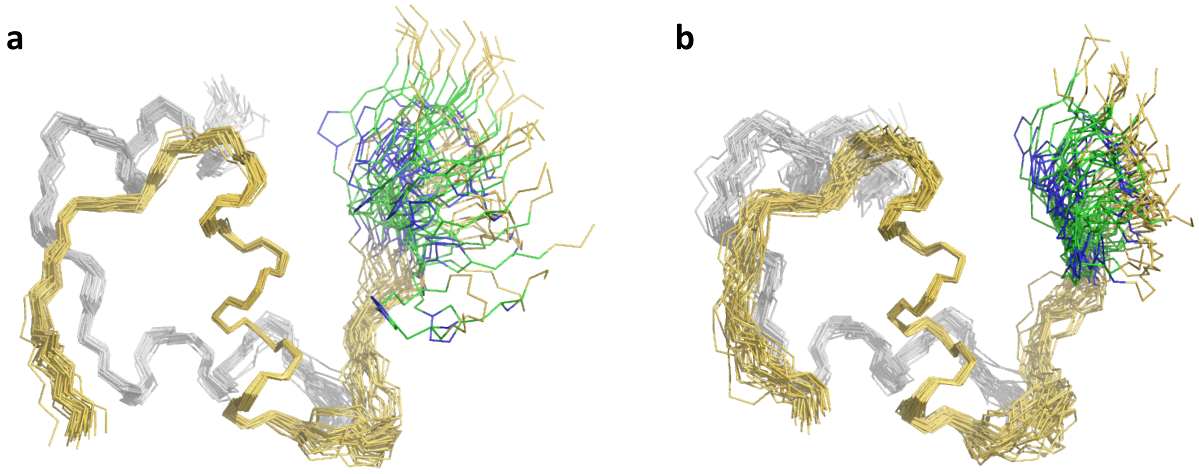

Supplementary Figure S6. Set of converged NMR structures for analogue 8. Set of converged NMR structures for analogue **8** at pH 1.9 **(a)** and pH 8.0 **(b)**. Insulin A chain is shown in gray, B chain is shown in yellow with the triazole cross-link at position B26-29 in green (carbon atoms) and blue (nitrogen atoms).

Supplementary Table S1. Structures of linear octapeptide precursors (1a-14a), cyclic octapeptide precursors (1b-14b) and respective resultant insulin analogues (1-14). **Prg** and **D-Prg** are L- or D-propargylglycine, Nle(εN$_3$) and D-Nle(εN$_3$) are L- or D-azidonorleucine, respectively. Nva(δN$_3$) is L-norvaline. Other non-natural substitutions (G for glycine and (*N*Me)A for *N*-methyl-alanine incorporated to C-terminal octapeptides are shown in red. DOI is *des*(B23-B30)octapeptide-insulin.

Code	Schematic structure	Structure
**1a**	G^{B23}-D-Prg-FYTP-Nle(εN$_3$)-T^{B30}	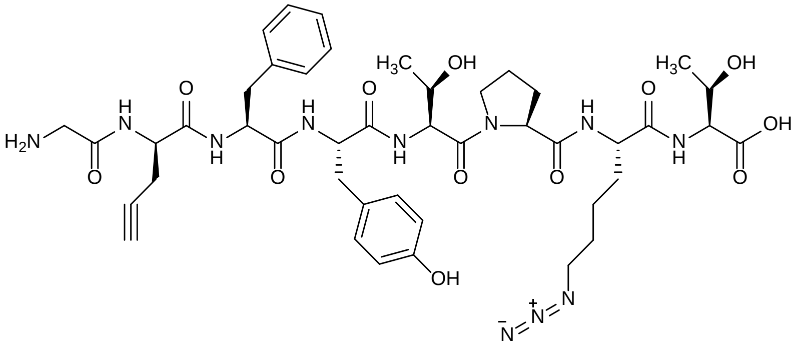
**1b**	Cyclo[G^{B23}-D-Prg-FYTP-Nle(εN$_3$)-T^{B30}]	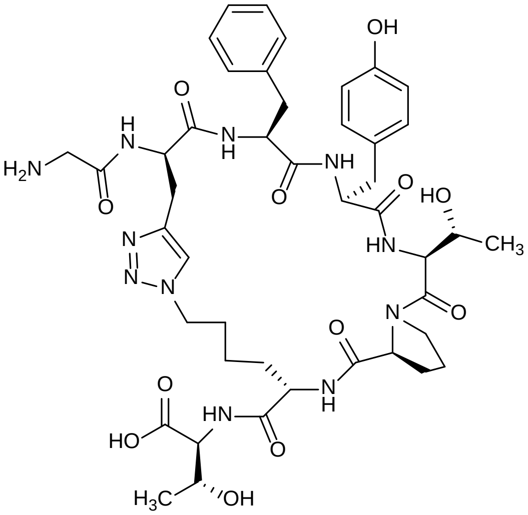

**1**	Cyclo[G^{B23}-D-Prg-FYTP-Nle(εN$_3$)-T^{B30}]-insulin	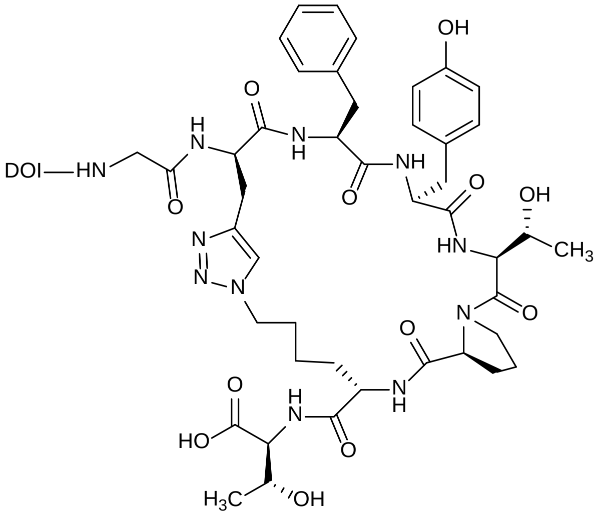
**2a**	G^{B23}-Prg-FYTP-Nle(εN$_3$)-T^{B30}	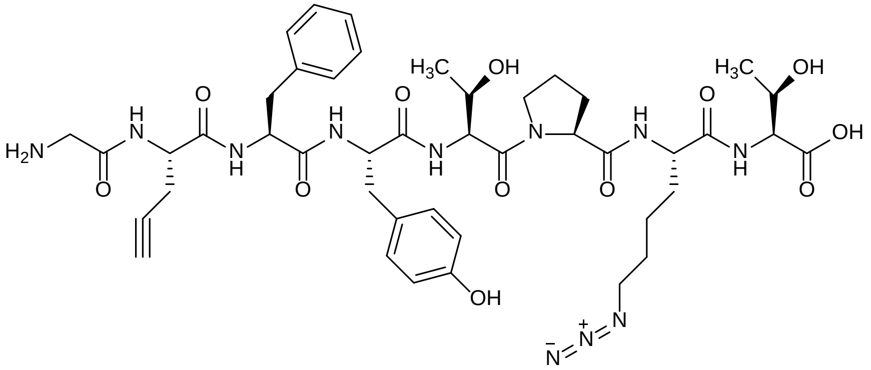
**2b**	Cyclo[G^{B23}-Prg-FYTP-Nle(εN$_3$)-T^{B30}]	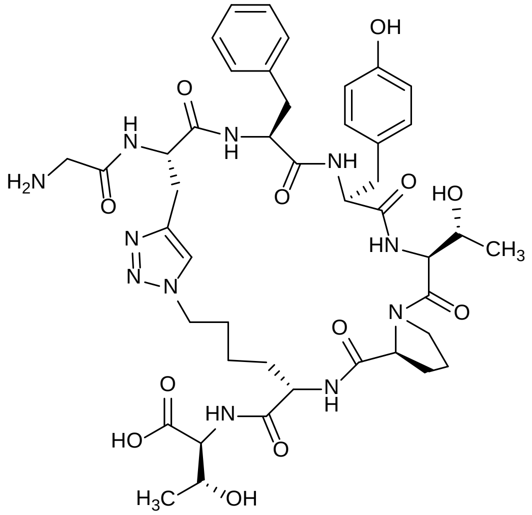

**2**	Cyclo[G^{B23}-Prg-FYTP-Nle(εN_3)-T^{B30}]-insulin	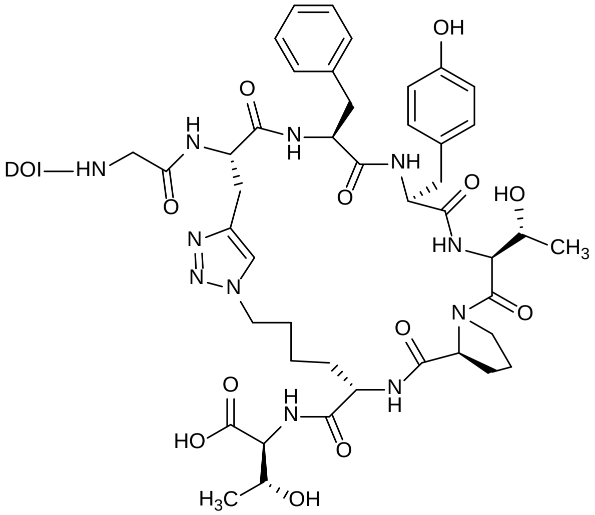
**3a**	G^{B23}-D-Nle(εN_3)-FYTP-Prg-T^{B30}	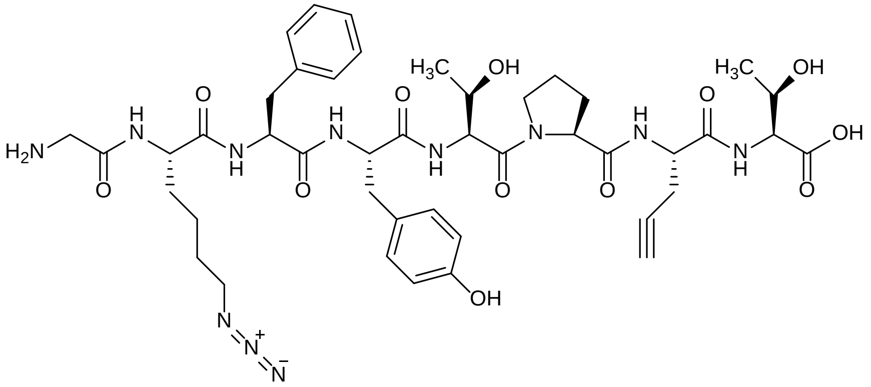
**3b**	Cyclo[G^{B23}-D-Nle(εN_3)-FYTP-Prg-T^{B30}]	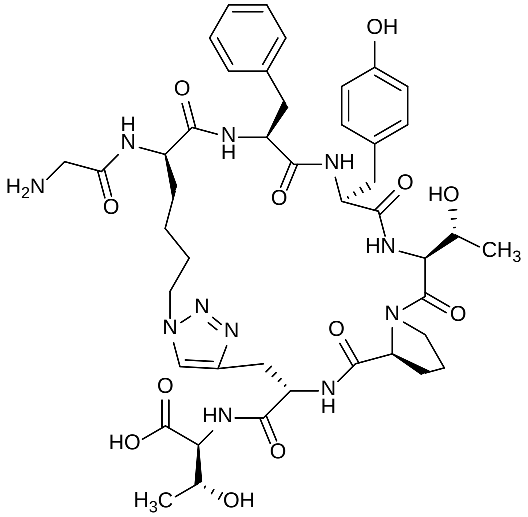

**3**	Cyclo[G^{B23}-D-Nle(εN$_3$)-FYTP-Prg-T^{B30}]-insulin	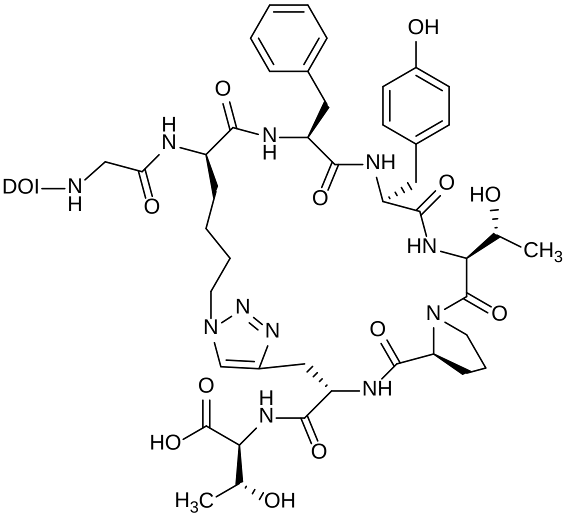
**4a**	G^{B23}-D-Prg-FYTP-D-Nle(εN$_3$)-T^{B30}	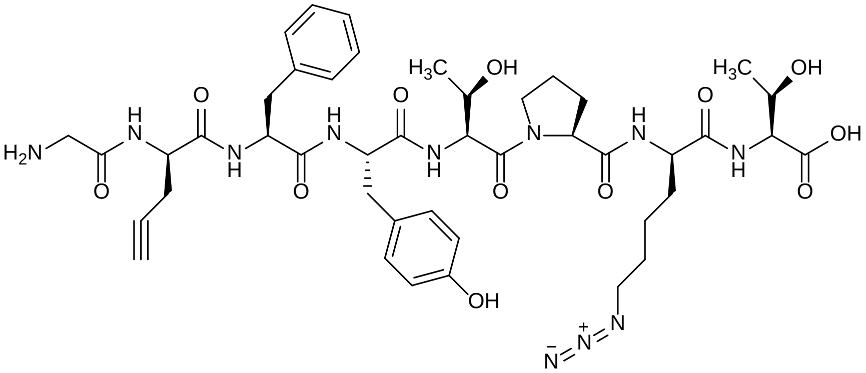
**4b**	Cyclo[G^{B23}-D-Prg-FYTP-D-Nle(εN$_3$)-T^{B30}]	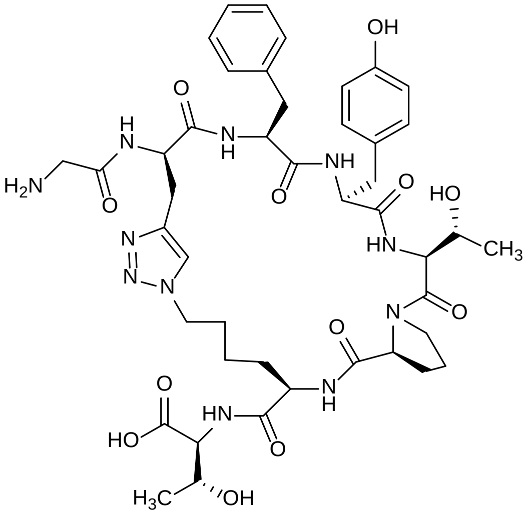

**4**	Cyclo[G^{B23}-D-Prg-FYTP-D-Nle(εN_3)-T^{B30}]-insulin	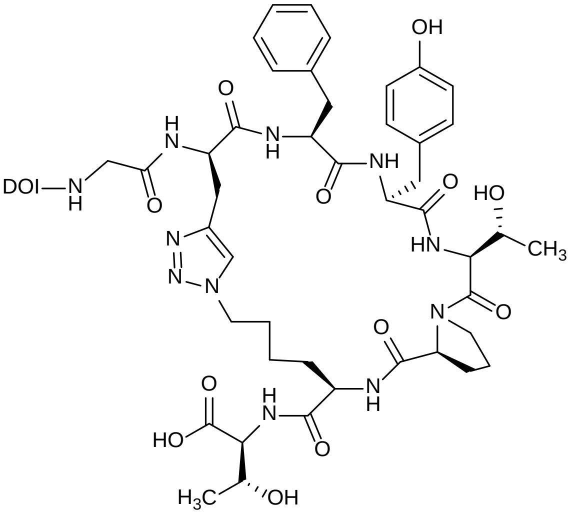
**5a**	G^{B23}-D-Nle(εN_3)-FYTP-D-Prg-T^{B30}	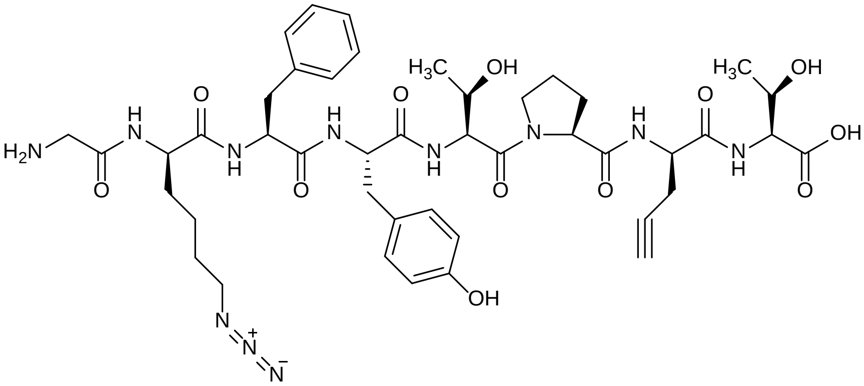
**5b**	Cyclo[G^{B23}-D-Nle(εN_3)-FYTP-D-Prg-T^{B30}]	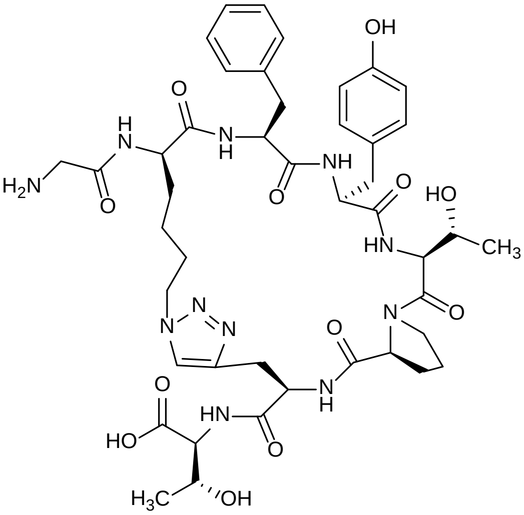

**5**	Cyclo[G^{B23}-D-Nle(εN_3)-FYTP-D-Prg-T^{B30}]-insulin	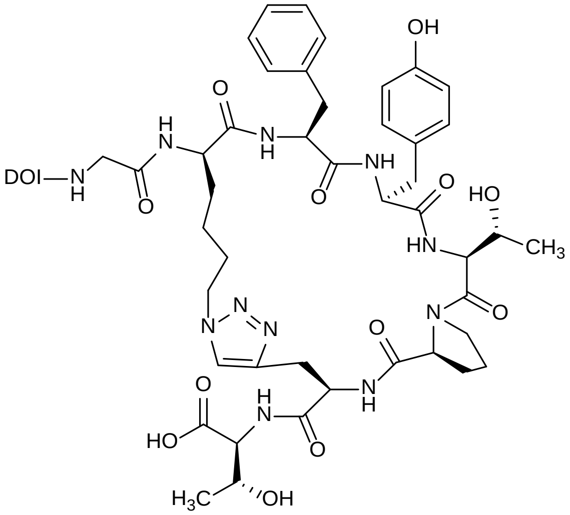
**6a**	G^{B23}F-Prg-YTP-Nle(εN_3)-T^{B30}	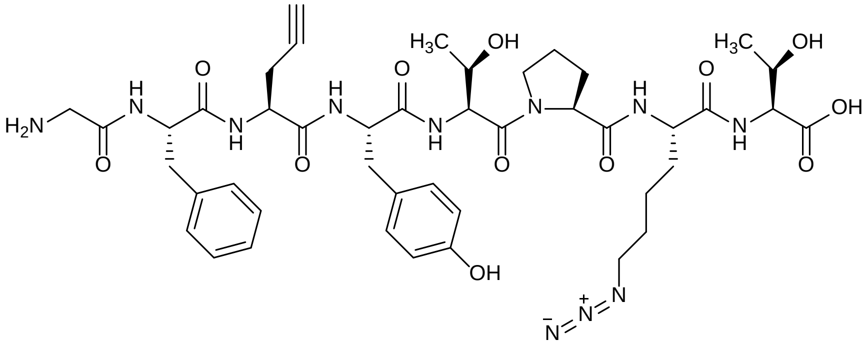
**6b**	Cyclo[G^{B23}F-Prg-YTP-Nle(εN_3)-T^{B30}]	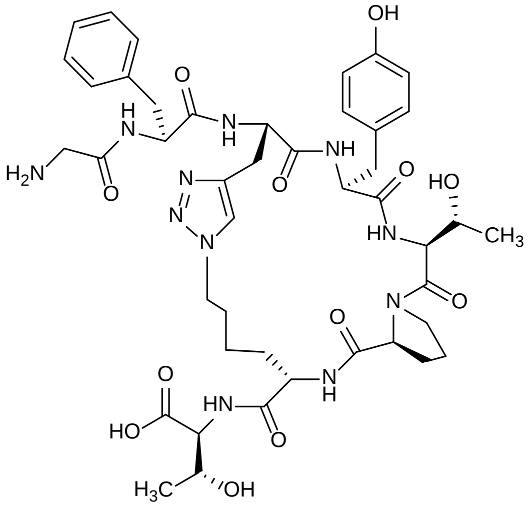

**6**	Cyclo[G^{B23}F-Prg-YTP-Nle(εN$_3$)-T^{B30}]-insulin	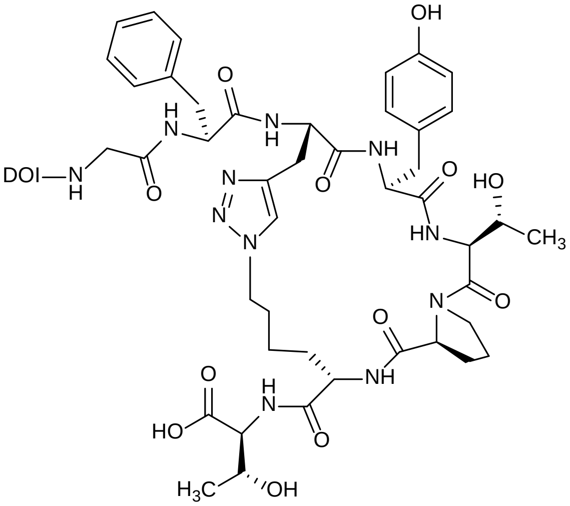
**7a**	G^{B23}F-D-Prg-YTP-Nle(εN$_3$)-T^{B30}	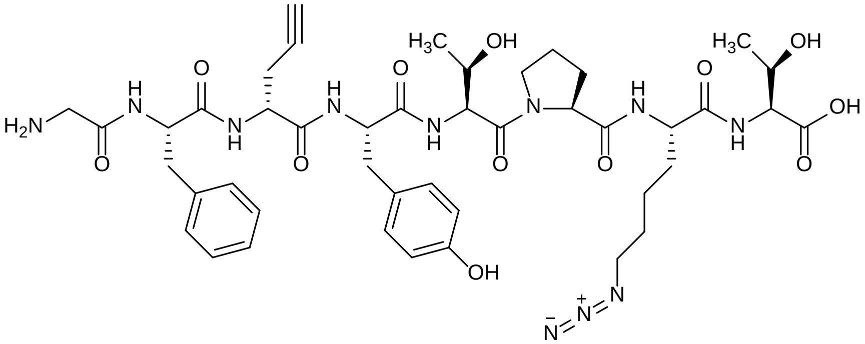
**7b**	Cyclo[G^{B23}F-D-Prg-YTP-Nle(εN$_3$)-T^{B30}]	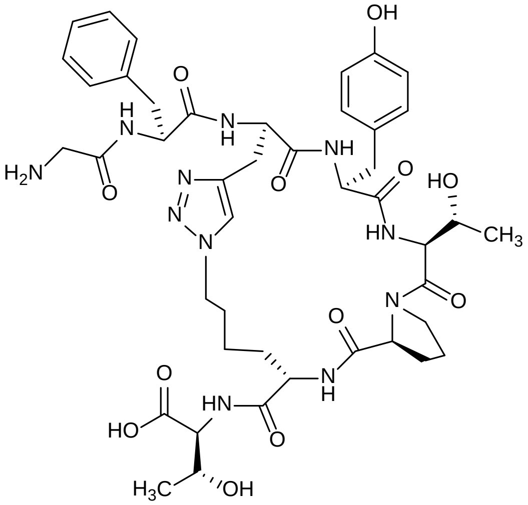

**7**	Cyclo[G^{B23}F-D-Prg-YTP-Nle(εN$_3$)-T^{B30}]-insulin	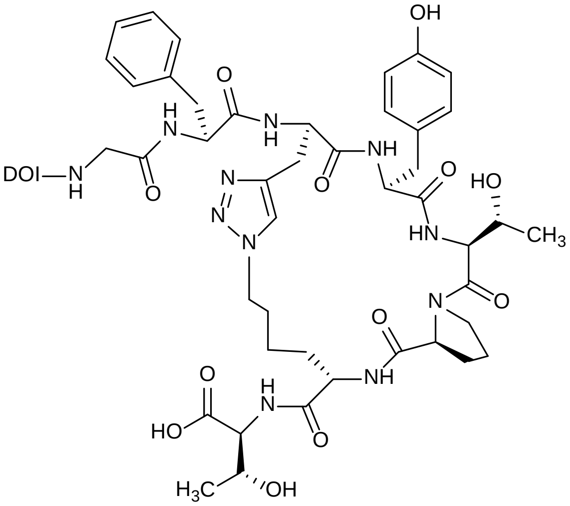
**8a**	G^{B23}FF-Nva(δN$_3$)-TP-Prg-T^{B30}	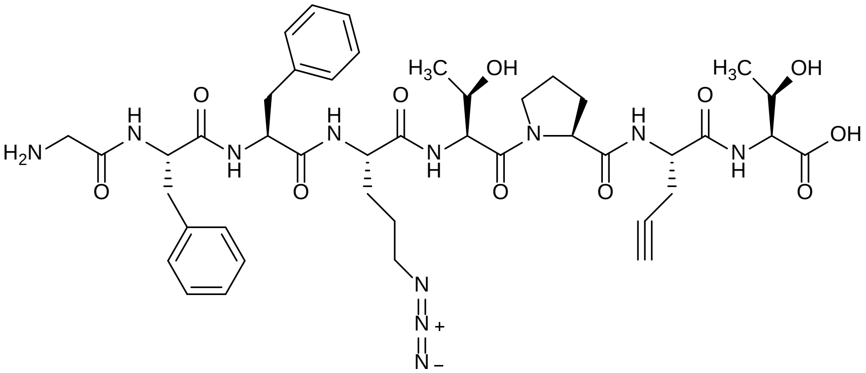
**8b**	Cyclo[G^{B23}FF-Nva(δN$_3$)-TP-Prg-T^{B30}]	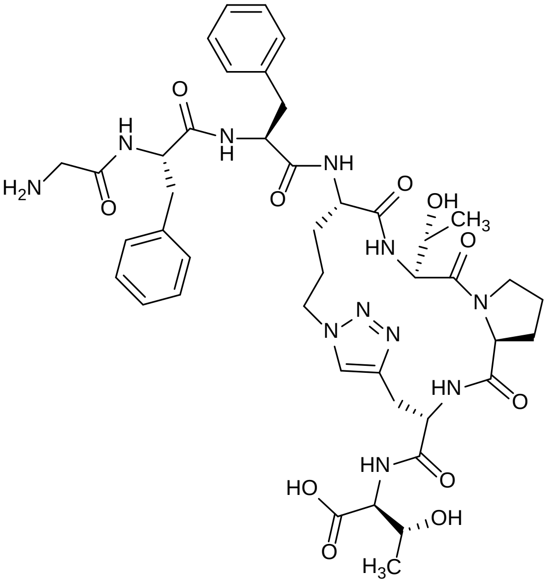

**8**	Cyclo[G^{B23}FF-Nva(δN_3)-TP-Prg-T^{B30}]-insulin	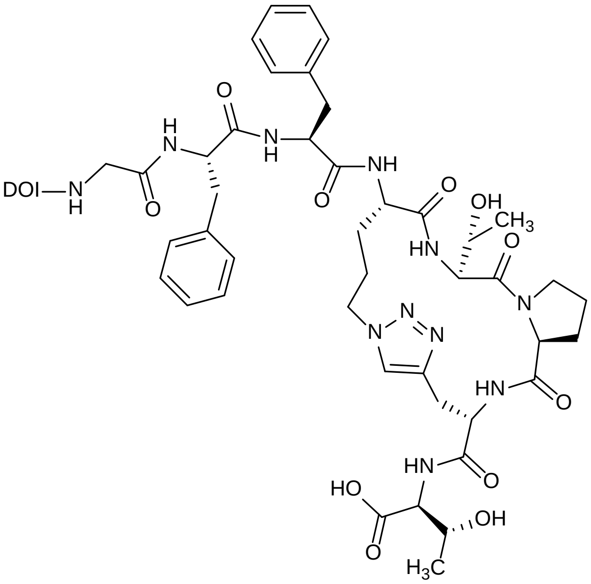
**9a**	G^{B23}FF-Prg-TP-Nva(δN_3)-T^{B30}	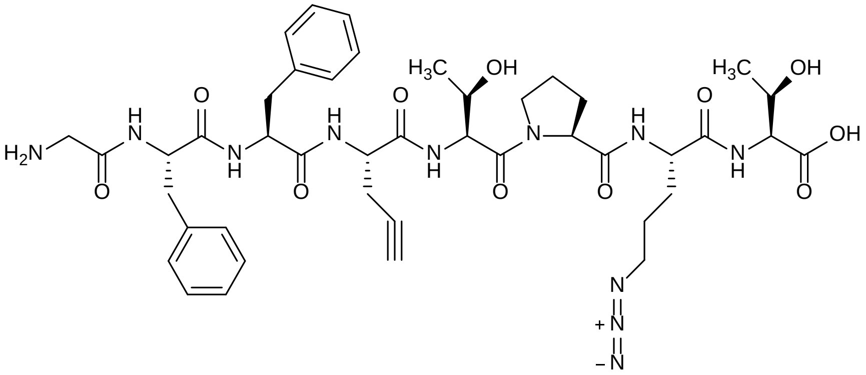
**9b**	Cyclo[G^{B23}FF-Prg-TP-Nva(δN_3)-T^{B30}]	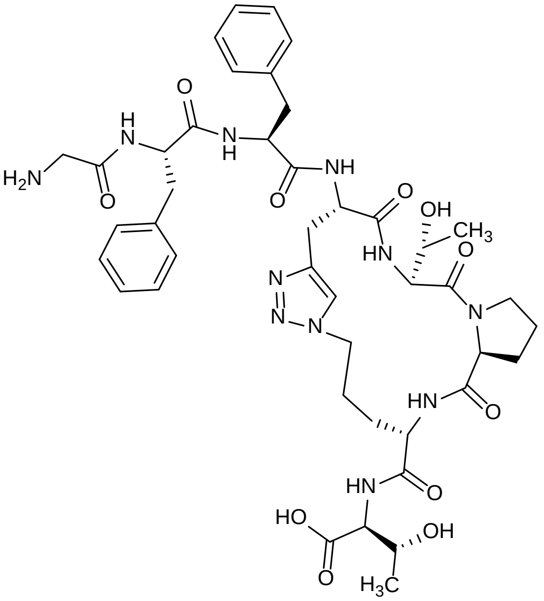

**9**	Cyclo[G^{B23}FF-Prg-TP-Nva(δN$_3$)-T^{B30}]-insulin	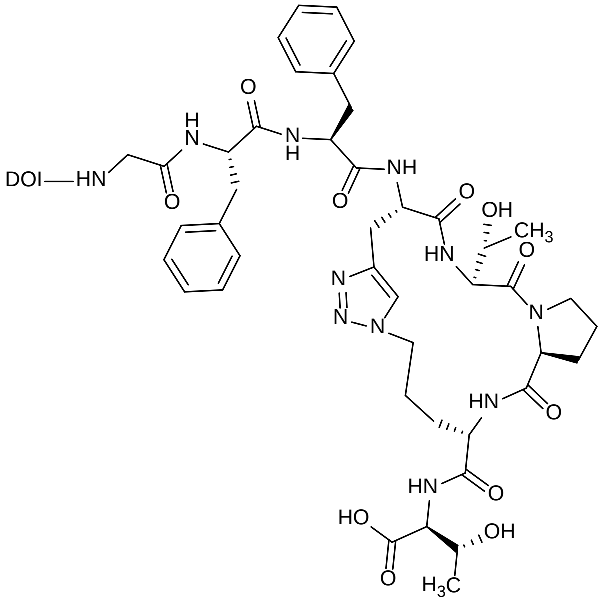
**10a**	G^{B23}FFY-Nva(δN$_3$)-P-Prg-T^{B30}	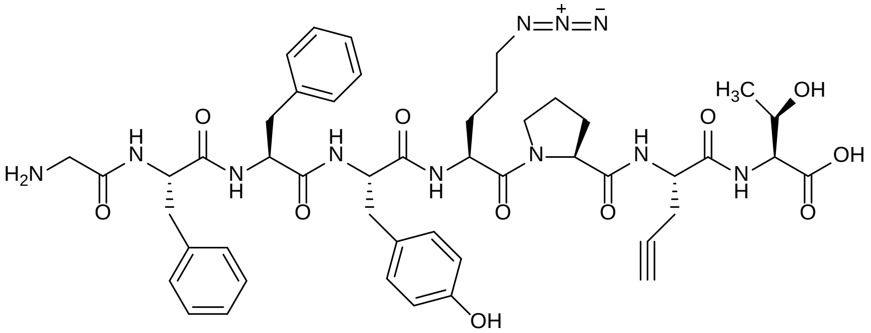
**10b**	Cyclo[G^{B23}FFY-Nva(δN$_3$)-P-Prg-T^{B30}]	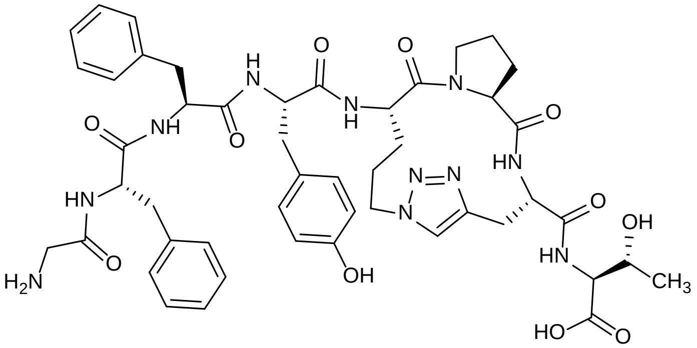
**10**	Cyclo[G^{B23}FFY-Nva(δN$_3$)-P-Prg-T^{B30}]-insulin	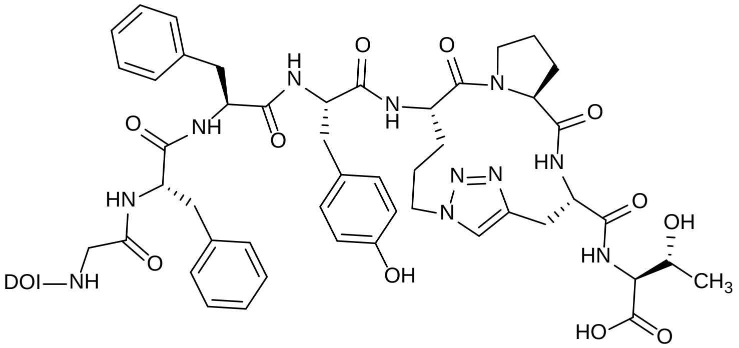

**11a**	G^{B23}FFY-Nva(δN$_3$)-P-G-Prg^{B30}	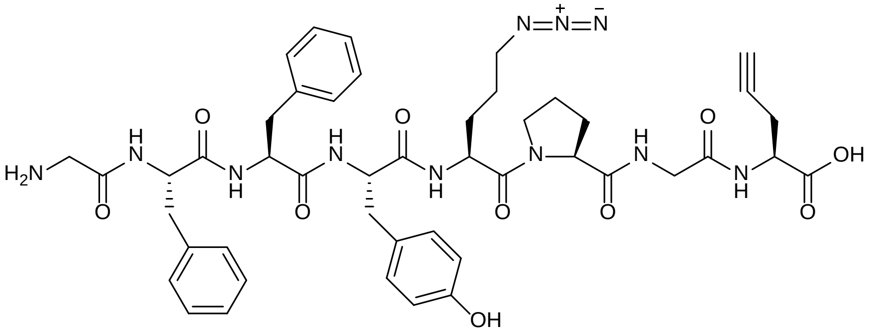
**11b**	Cyclo[G^{B23}FFY-Nva(δN$_3$)-P-G-Prg^{B30}]	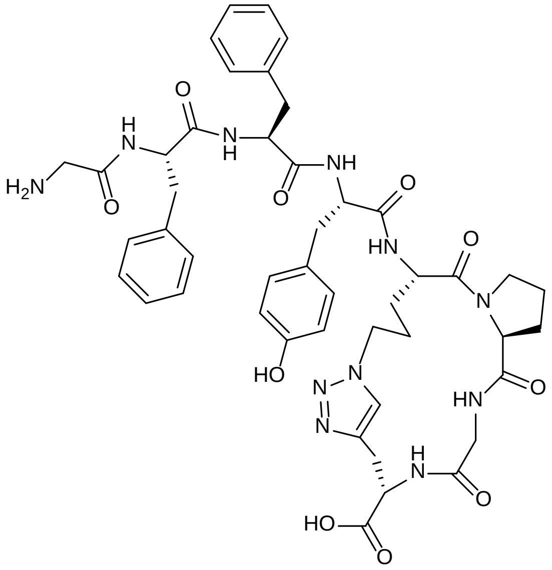
**11**	Cyclo[G^{B23}FFY-Nva(δN$_3$)-P-G-Prg^{B30}]-insulin	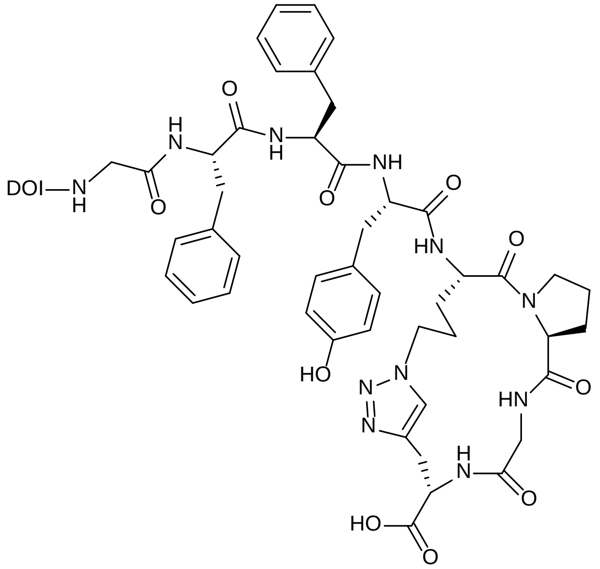

**12a**	G^{B23}-G-FY-Nva(δN_3)-P-Prg-T^{B30}	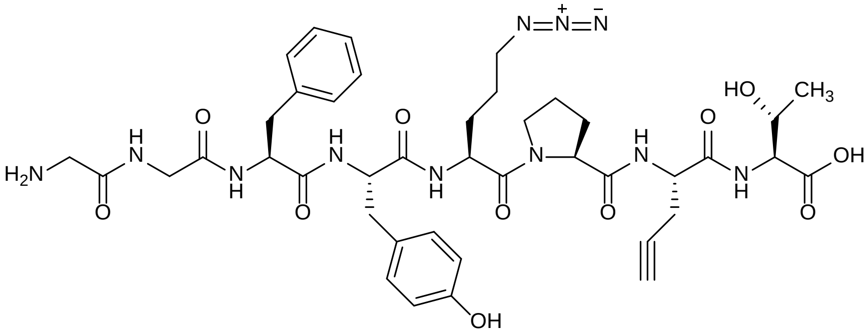
**12b**	Cyclo[G^{B23}-G-FY-Nva(δN_3)-P-Prg-T^{B30}]	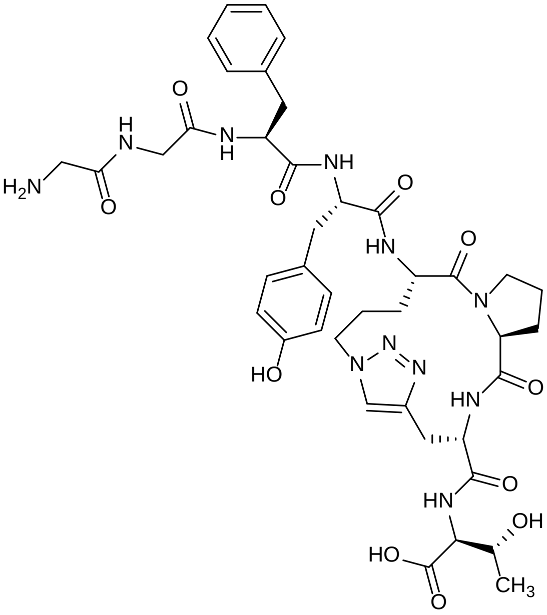
**12**	Cyclo[G^{B23}-G-FY-Nva(δN_3)-P-Prg-T^{B30}]-insulin	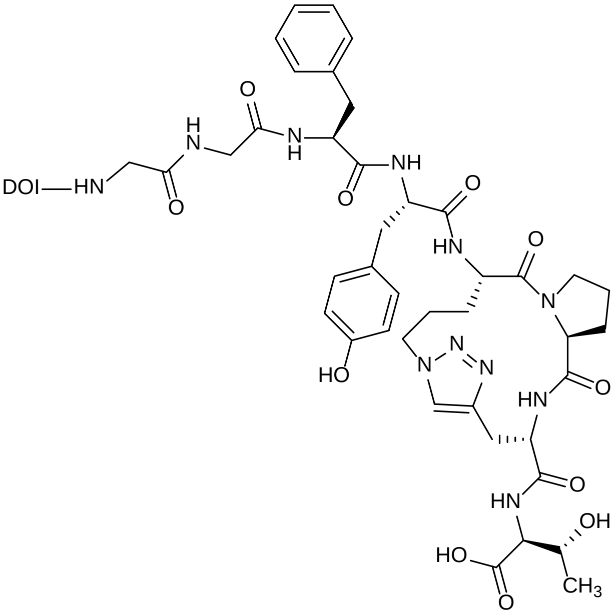

**13a**	G^{B23}-G-F-Nva(δN_3)-TP-Prg-T^{B30}	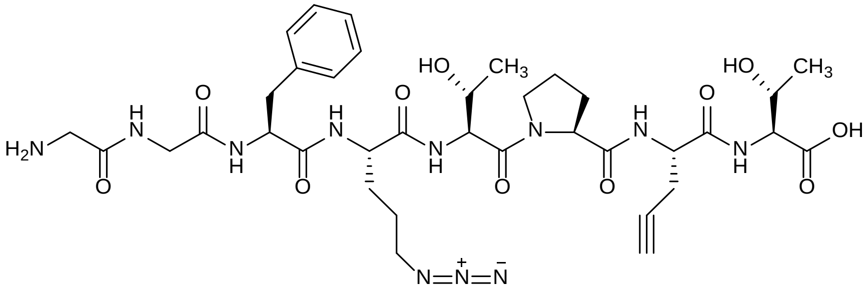
**13b**	Cyclo[G^{B23}-G-F-Nva(δN_3)-TP-Prg-T^{B30}]	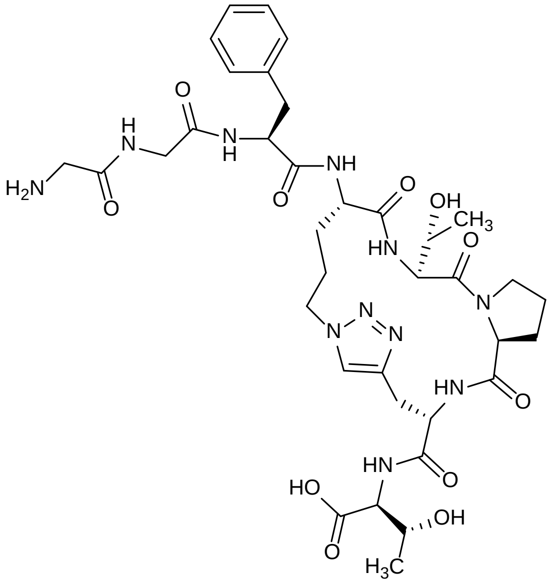
**13**	Cyclo[G^{B23}-G-F-Nva(δN_3)-TP-Prg-T^{B30}]-insulin	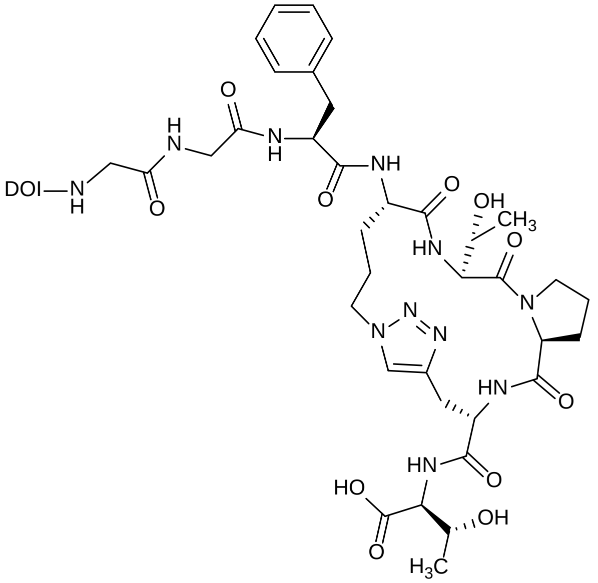

**14a**	G^{B23}-G-F-(*N*Me)A-Nva(δN_3)-P-Prg-T^{B30}	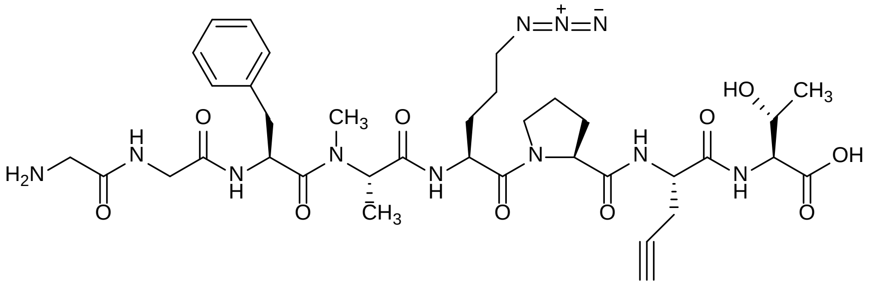
**14b**	Cyclo[G^{B23}-G-F-(*N*Me)A-Nva(δN_3)-P-Prg-T^{B30}]	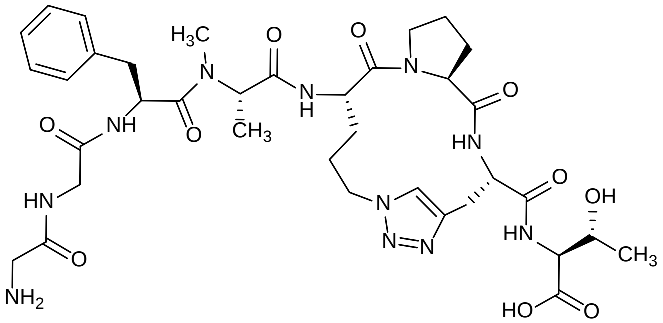
**14**	Cyclo[G^{B23}-G-F-(*N*Me)A-Nva(δN_3)-P-Prg-T^{B30}]-insulin	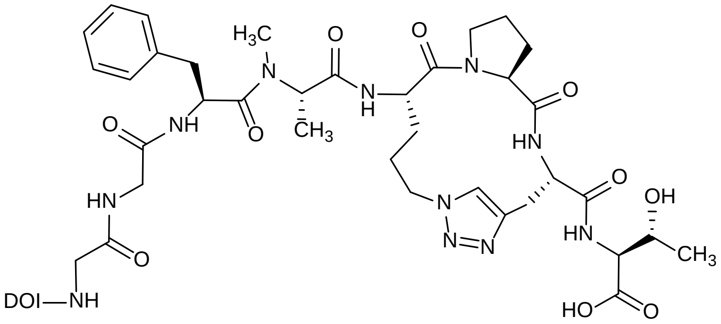

Synthetic yields and characterization data for octapeptides and insulin analogues.

The cyclic octapeptides **1b-13b** were prepared from respective linear precursor **1a-13a** by the cycloaddition method A. The cyclic octapeptide **14b** was prepared from **14a** by the method B (see Methods in the main text).

Linear octapeptide 1a. The yield (RP-HPLC) was 57% (133 mg; 142 μmol). HRMS-ESI (m/z): $[MH]^+$ calcd. $C_{44}H_{60}N_{11}O_{12}$, for 934.4417; found 934.4416.

Cyclic octapeptide 1b. The compound was prepared from **1a**. The yield (RP-HPLC) was 82 % (41 mg; 44 μmol). MS-ESI (m/z): $[MH]^+$ calcd. for $C_{44}H_{60}N_{11}O_{12}$ 934.44; found 934.44.

Insulin analogue 1. Analogue **2** was prepared from DOI and **1b**. The yield (relative to DOI as the limiting compound of the reaction) after RP-HPLC purification was 4 % (1.4 mg; 0.24 μmol). HRMS-ESI (m/z): $[MH]^+$ calcd. 5778.60; found 5778.60.

Linear octapeptide 2a. The yield (crude peptide) was cca 96% (225 mg; cca 241 μmol). MS-ESI (m/z): $[MH]^+$ calcd. for $C_{44}H_{60}N_{11}O_{12}$ 934.44; found 934.44.

Cyclic octapeptide 2b. The compound was prepared from crude linear octapeptide **2a** (160 mg; cca 171 μmol). The yield (RP-HPLC) was cca 26 % (42 mg; cca 45 μmol). MS-ESI (m/z): $[MH]^+$ calcd. for $C_{44}H_{60}N_{11}O_{12}$ 934.44; found 934.44.

Insulin analogue 2. Analogue **2** was prepared from DOI and **2b**. The yield (RP-HPLC) was 15 % (5.4 mg; 0.93 μmol). HRMS-ESI (m/z): $[MH]^+$ calcd. 5778.60; found 5778.61.

Linear octapeptide 3a. The yield (RP-HPLC) was 33% (77 mg; 83 μmol). MS-ESI (m/z): $[MH]^+$ calcd. for $C_{44}H_{60}N_{11}O_{12}$ 934.44; found 934.44.

Cyclic octapeptide 3b. The compound was prepared from 3**a**. The yield (RP-HPLC) was 88 % (44 mg; 47 μmol). MS-ESI (m/z): $[MH]^+$ calcd. for $C_{44}H_{60}N_{11}O_{12}$ 934.44; found 934.44.

Insulin analogue 3. Analogue **3** was prepared from DOI and **3b**. The yield (RP-HPLC) was 5 % (1.8 mg; 0.31 μmol). HRMS-ESI (m/z): $[MH]^+$ calcd. 5778.60; found 5778.61.

Linear octapeptide 4a. The yield (RP-HPLC) was 55% (129 mg; 138 μmol). MS-ESI (m/z): $[MH]^+$ calcd. for $C_{44}H_{60}N_{11}O_{12}$ 934.44; found 934.44.

Cyclic octapeptide 4b. The compound was prepared from **4a**. The yield (RP-HPLC) was 62 % (31 mg; 33 μmol). MS-ESI (m/z): $[MH]^+$ calcd. for $C_{44}H_{60}N_{11}O_{12}$ 934.44; found 934.44.

Insulin analogue 4. Analogue **4** was prepared from DOI and **4b**. The yield (RP-HPLC) was 8 % (2.6 mg; 0.45 μmol). HRMS-ESI (m/z): $[MH]^+$ calcd. 5778.60; found 5778.62.

Linear octapeptide 5a. The yield (RP-HPLC) was 60% (140 mg; 150 μmol). MS-ESI (m/z): $[MH]^+$ calcd. for $C_{44}H_{60}N_{11}O_{12}$ 934.44; found 934.44.

Cyclic octapeptide 5b. The compound was prepared from **5a**. The yield (RP-HPLC) was 62 % (31 mg; 33 μmol). MS-ESI (m/z): $[MH]^+$calcd. for $C_{44}H_{60}N_{11}O_{12}$ 934.44; found 934.44.

Insulin analogue 5. Analogue **5** was prepared from DOI and **5b.**. The yield (RP-HPLC) was 5 % (1.8 mg; 0.31 μmol). HRMS-ESI (m/z): $[MH]^+$ calcd. 5778.60; found 5778.61.

Linear octapeptide 6a. The yield (RP-HPLC) was 47% (109 mg; 117 μmol). HRMS-ESI (m/z): $[MH]^+$ calcd. for $C_{44}H_{60}N_{11}O_{12}$ 934.4417; found 934.4409, $[MNa]^+$ calcd. for $C_{44}H_{59}N_{11}O_{12}Na$ 956.4237; found 956.4228.

Cyclic octapeptide 6b. The compound was prepared from **6a**. The yield (RP-HPLC) was 74 % (37 mg; 33 μmol). HRMS-ESI (m/z): $[MH]^+$ calcd for $C_{44}H_{60}N_{11}O_{12}$ 934.4417; found 934.4429, $[MNa]^+$ calcd for $C_{44}H_{59}N_{11}O_{12}Na$ 956.4237; found 956.4247.

Insulin analogue 6. The analogue **6** was prepared from DOI and **6b**. The yield (RP-HPLC) purification was 7 % (2.3 mg; 0.40 μmol). HRMS-ESI (m/z): $[MH]^+$ calcd. 5778.60; found 5778.60.

Linear octapeptide 7a. The yield (RP-HPLC) was 37% (86 mg; 92 μmol). HRMS-ESI (m/z): $[MH]^+$ calcd. for 934.4417 $C_{44}H_{60}N_{11}O_{12}$; found 934.4403.

Cyclic octapeptide 7b. The compound was prepared from **7a**. The yield (RP-HPLC) was 74 % (37 mg; 33 μmol). HRMS-ESI (m/z): $[MH]^+$ calcd. for $C_{44}H_{60}N_{11}O_{12}$ 934.4417; found 934.4423.

Insulin analogue 7. The analogue **7** was prepared from DOI and **7b**. The yield (RP-HPLC) was 30 % (10 mg; 1.73 μmol). HRMS-ESI (m/z): $[MH]^+$ calcd. 5778.60, found 5778.60.

Linear octapeptide 8a. The yield (RP-HPLC) was 63% (143 mg; 158 μmol). HRMS-ESI (m/z): $[MH]^+$ calcd. for $C_{43}H_{58}N_{11}O_{11}$ 904.4312; found 904.4318.

Cyclic octapeptide 8b. The compound was prepared from **8a**. The yield (RP-HPLC) was 40 % (20 mg; 22 μmol). HRMS-ESI (m/z): $[MH]^+$ calcd. for $C_{43}H_{58}N_{11}O_{11}$ 904.4312; found 904.4317.

Insulin analogue 8. Analogue **8** was prepared from DOI and **8b.**. The yield (RP-HPLC) was 9.8 % (2.4 mg; 0.42 μmol). HRMS-ESI (m/z): $[MH]^+$ calcd. 5748.59; found 5748.59.

Linear octapeptide 9a. The yield (RP-HPLC) was 48% (109 mg; 120 μmol). HRMS-ESI (m/z): $[MH]^+$ calcd. for $C_{43}H_{58}N_{11}O_{11}$ 904.4312; found 904.4317.

Cyclic octapeptide 9b. The compound was prepared from **9a**. The yield (RP-HPLC) was 45 % (23 mg; 25 μmol). HRMS-ESI (m/z): $[MH]^+$ calcd. for $C_{43}H_{58}N_{11}O_{11}$ 904.4312; found 904.4316.

Insulin analogue 9. Analogue **9** was prepared from DOI and **9b**. The yield (RP-HPLC) was 9.8 % (2.9 mg; 0.51 μmol). HRMS-ESI (m/z): $[MH]^+$ calcd. 5748.59; found 5748.59.

Linear octapeptide 10a. The yield (crude peptide) was cca 72% (175 mg; cca 181 μmol). HR-ESI (m/z): $[MH]^+$ calcd. for $C_{48}H_{60}N_{11}O_{11}$ 966.4468; found 966.4467.

Cyclic octapeptide 10b. The compound was prepared from 160 mg (cca 166 μmol) of crude linear octapeptide **10a**. The yield (RP-HPLC) was cca 37 % (60 mg; cca 62 μmol). HRMS-ESI (m/z): $[MH]^+$ calcd. for $C_{43}H_{58}N_{11}O_{11}$ 966.4468; found 966.4469.

Insulin analogue 10. Analogue **10** was prepared from DOI and **10b**. The yield (RP-HPLC) was 13 % (2.4 mg; 0.41 μmol). HR-ESI $[MH]^+$ calculated 5810.61, found 5810.62.

Linear octapeptide 11a. The yield (RP-HPLC) was 33% (77 mg; 120 μmol). MS-ESI (m/z): $[MH]^+$ calcd. for $C_{46}H_{56}N_{11}O_{10}$ 922.42; found 922.42.

Cyclic octapeptide 11b. The compound was prepared from **11a**. The yield (RP-HPLC) was 50 % (25 mg; 27 μmol). MS-ESI (m/z): $[MH]^+$ calcd. for $C_{46}H_{56}N_{11}O_{10}$ 922.42; found 922.42.

Insulin analogue 11. Analogue **11** was prepared from DOI and **11b**. The yield (RP-HPLC) was 14 % (4.9 mg; 0.84 μmol). HRMS-ESI $[MH]^+$ calcd. 5766.58; found 5766.58.

Linear octapeptide 12a. The yield (RP-HPLC) was 45% (99 mg; 113 μmol). HRMS-ESI (m/z): $[MH]^+$ calcd. for $C_{41}H_{54}N_{11}O_{11}$ 876.4004; found 876.4004, $[MNa]^+$ calcd. for $C_{41}H_{53}N_{11}O_{11}Na$ 898.3824; found 898.3818.

Cyclic octapeptide 12b. The compound was prepared from **12a**. The yield (RP-HPLC) was 52 % (26 mg; 30 μmol). HRMS-ESI (m/z): $[MH]^+$ calcd. for $C_{41}H_{54}N_{11}O_{11}$ 876.4004; found 876.4004, $[MNa]^+$ calcd. for $C_{41}H_{53}N_{11}O_{11}Na$ 898.3824; found 898.3818.

Insulin analogue 12. Analogue **12** was prepared from DOI and **12b**. The yield (RP-HPLC) was 5 % (1.7 mg; 0.30 μmol). HRMS-ESI (m/z): $[MH]^+$ calcd. 5720.56; found 5720.57.

Linear octapeptide 13a. The yield (RP-HPLC) was 26 % (52 mg; 64 μmol). HRMS-ESI (m/z): $[MH]^+$ calcd. for $C_{36}H_{52}N_{11}O_{11}$ 814.3848; found 814.3838.

Cyclic octapeptide 13b. The compound was prepared from **13a**. The yield (RP-HPLC) was 36 % (18 mg; 22 μmol). MS-ESI (m/z): $[MH]^+$ calcd. for $C_{36}H_{52}N_{11}O_{11}$ 814.38; found 814.38.

Insulin analogue 13. Analogue **13** was prepared from DOI and **13b**. The yield (RP-HPLC) was 17 % (4.4 mg; 0.77 μmol). HRMS -ESI (m/z): $[MH]^+$ calcd. 5658.55; found 5658.55.

Linear octapeptide 14a. The yield (RP-HPLC) was 46 % (91 mg; 114 μmol). HRMS-ESI (m/z): $[MH]^+$ calcd. for $C_{36}H_{52}N_{11}O_{10}$ 798.3899; found 798.3893.

Cyclic octapeptide 14b. The compound was prepared from **14a**. The yield (RP-HPLC) was 49 % (25 mg; 31 μmol). HRMS-ESI (m/z): $[MH]^+$ calcd. for $C_{36}H_{52}N_{11}O_{10}$ 798.3899; found 798.3887.

Insulin analogue 14. Analogue **14** was prepared from DOI and **14b**. The yield (RP-HPLC) was 18 % (5.6 mg; 0.99 μmol). HRMS-ESI (m/z): $[MH]^+$ calcd. 5642.55; found 5642.55.

HPLC purification of octapeptides and insulin analogues.

Except octapeptides **2a** and **10a**, which were sufficiently pure in their crude forms, all the linear and cyclic octapeptides were purified using reversed-phase high-performance liquid chromatography (RP-HPLC) on Nucleosil C18 column (250 mm × 21 mm, 5 μm), at 9 ml/min, in a gradient of acetonitrile in water supplemented with 0.1% (v/v) TFA.

Target insulin analogues **1-14** were purified on Nucleosil C18 column (250 mm × 8 mm, 5 μm), at 3 ml/min, using a following gradient of acetonitrile (ACN) in water (v/v) supplemented with 0.1% (v/v) TFA: 0 min/8% ACN, 1 min/28% ACN, 21 min/36% ACN, 34 min/44% ACN, 36-37 min/72% ACN, 37.1 min/8% ACN.

The purity of analogues **1-14** was controlled by RP-HPLC on Nucleosil C18 column (250 mm × 4 mm, 5 μm) using the same gradient as above but at 1 ml/min. The absorbance was monitored at 218 nm. The RP-HPLC chromatograms of analogues **1-14** are shown in Figures S7-S20.

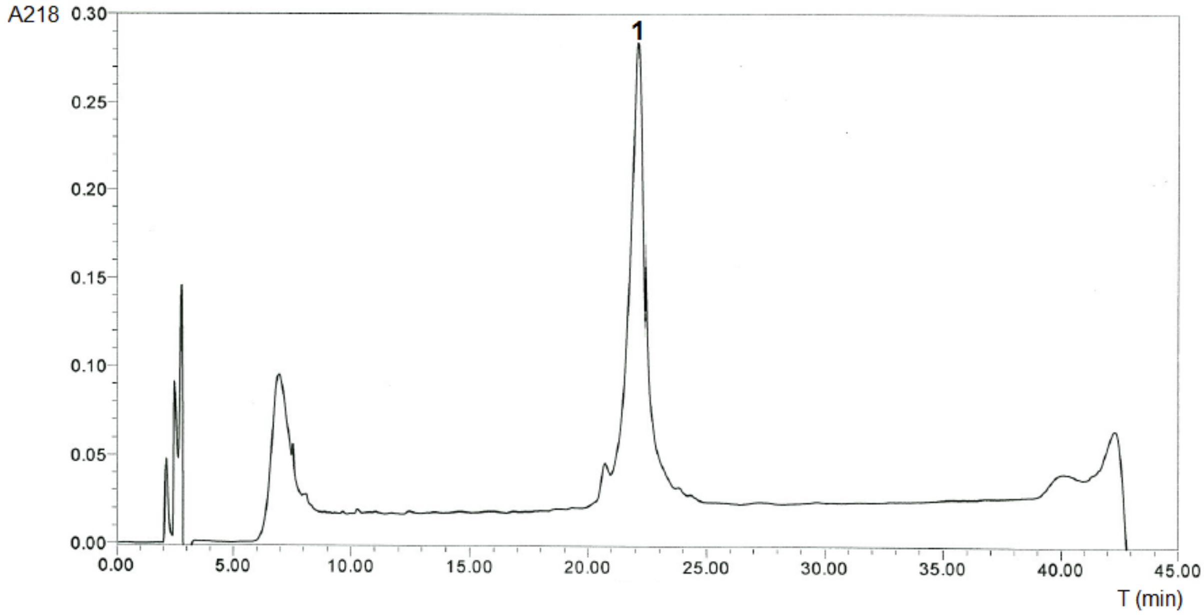

Figure S7. RP-HPLC analysis of analogue 1. The peak of the compound is marked by **1**.

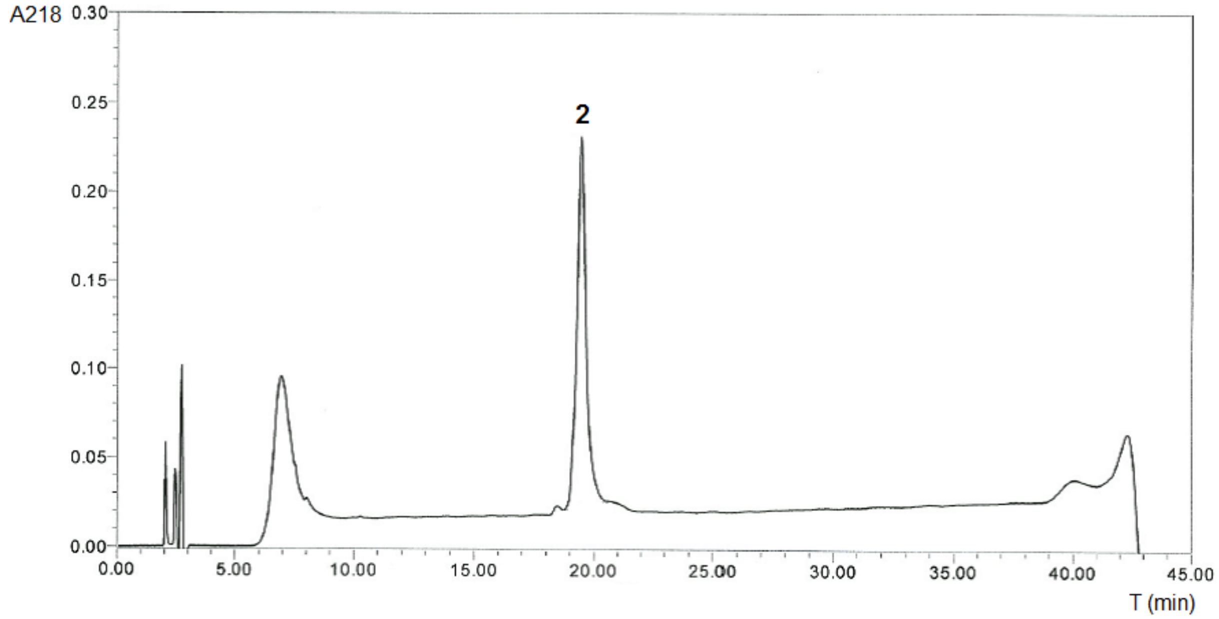

Figure S8. RP-HPLC analysis of analogue 2. The peak of the compound is marked by **2**.

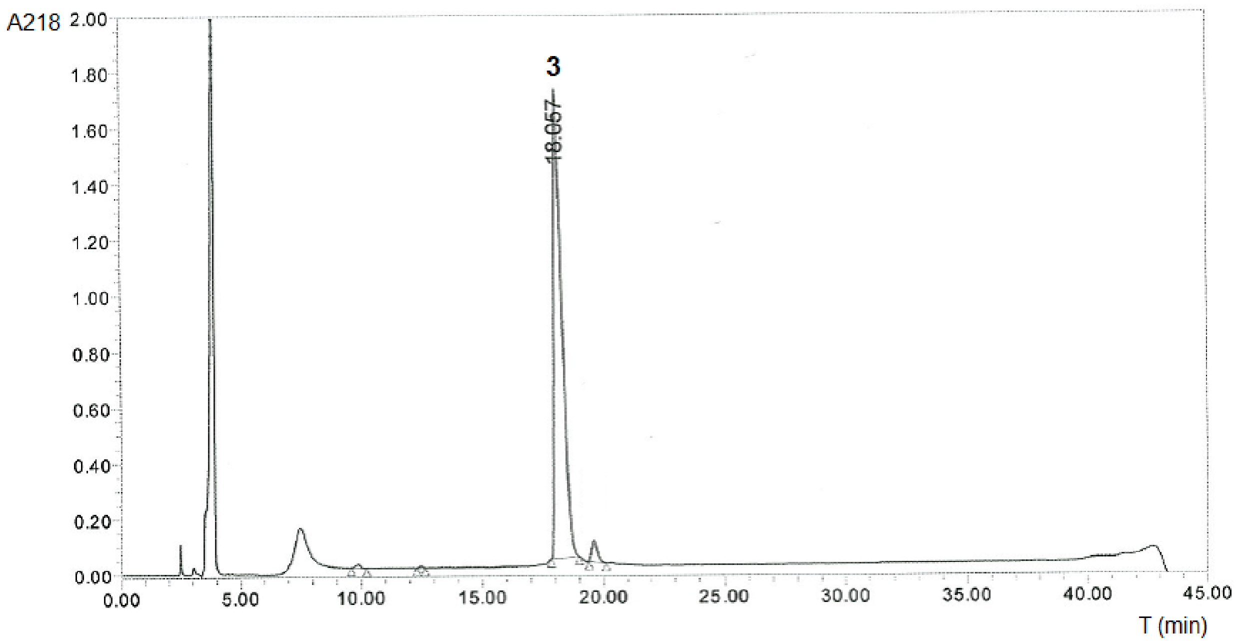

Figure S9. RP-HPLC analysis of analogue 3. The peak of the compound is marked by **3**.

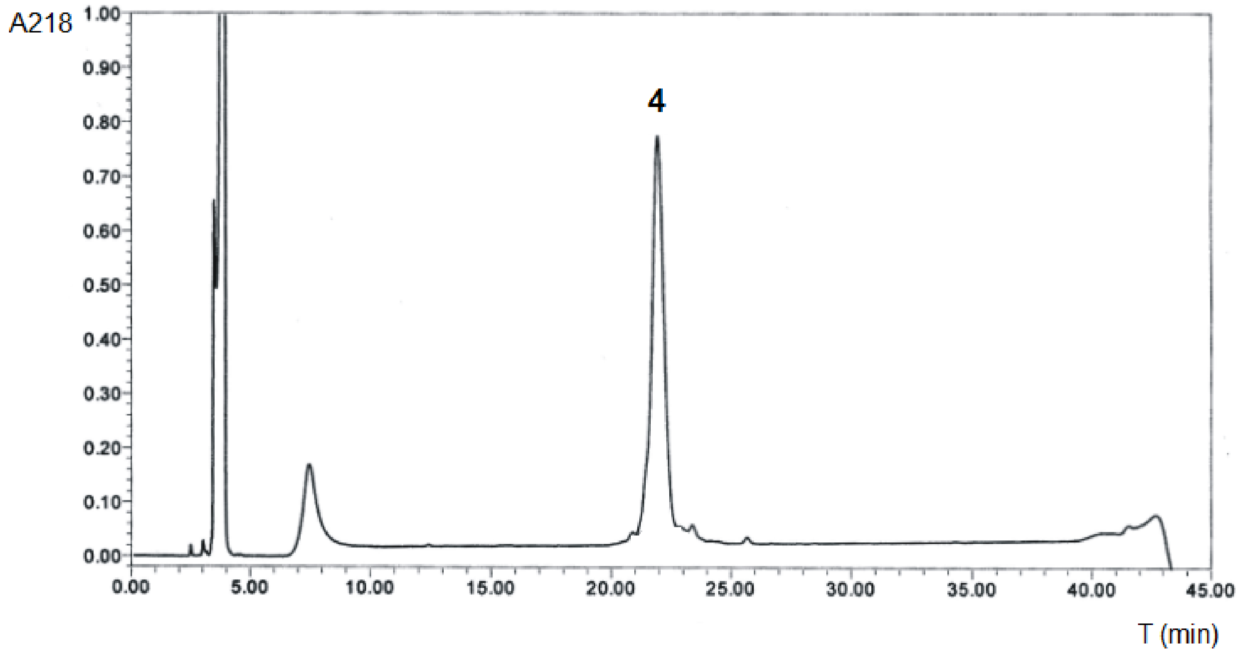

Figure S10. RP-HPLC analysis of analogue 4. The peak of the compound is marked by **4**.

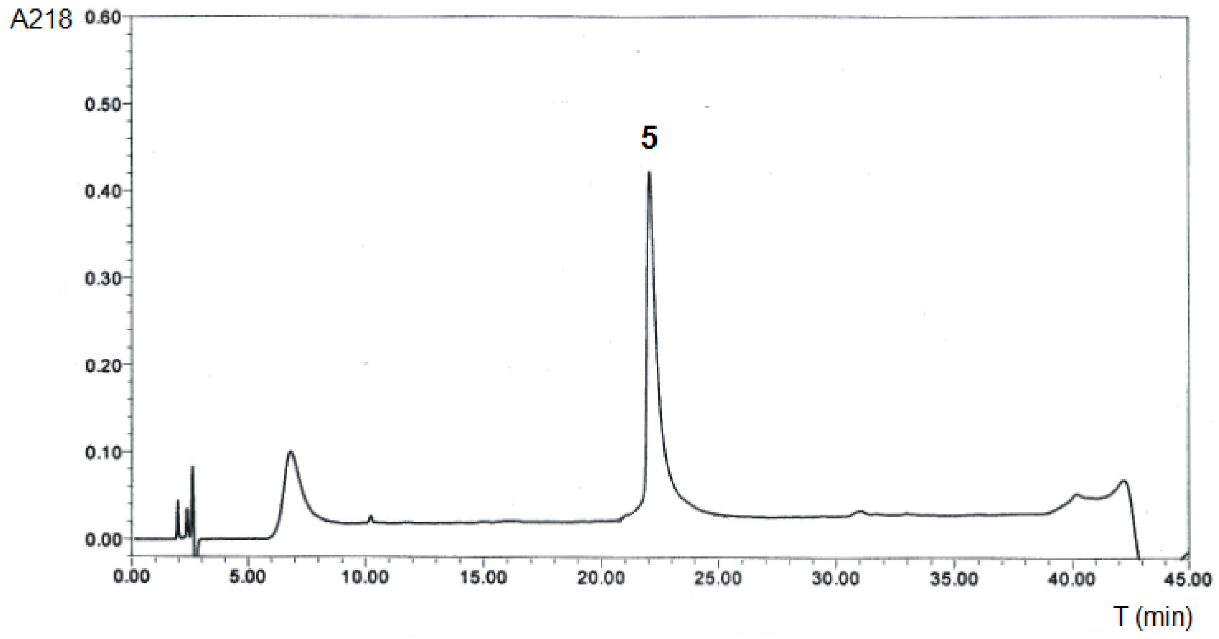

Figure S11. RP-HPLC analysis of analogue 5. The peak of the compound is marked by **5**.

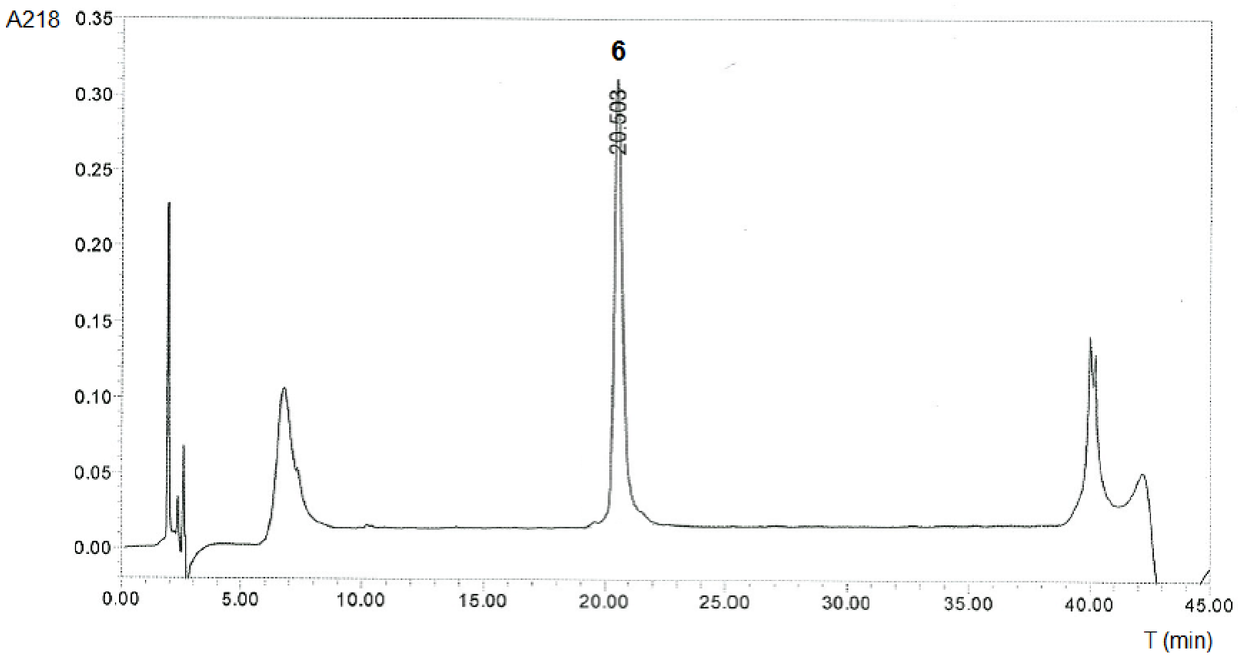

Figure S12. RP-HPLC analysis of analogue 6. The peak of the compound is marked by **6**.

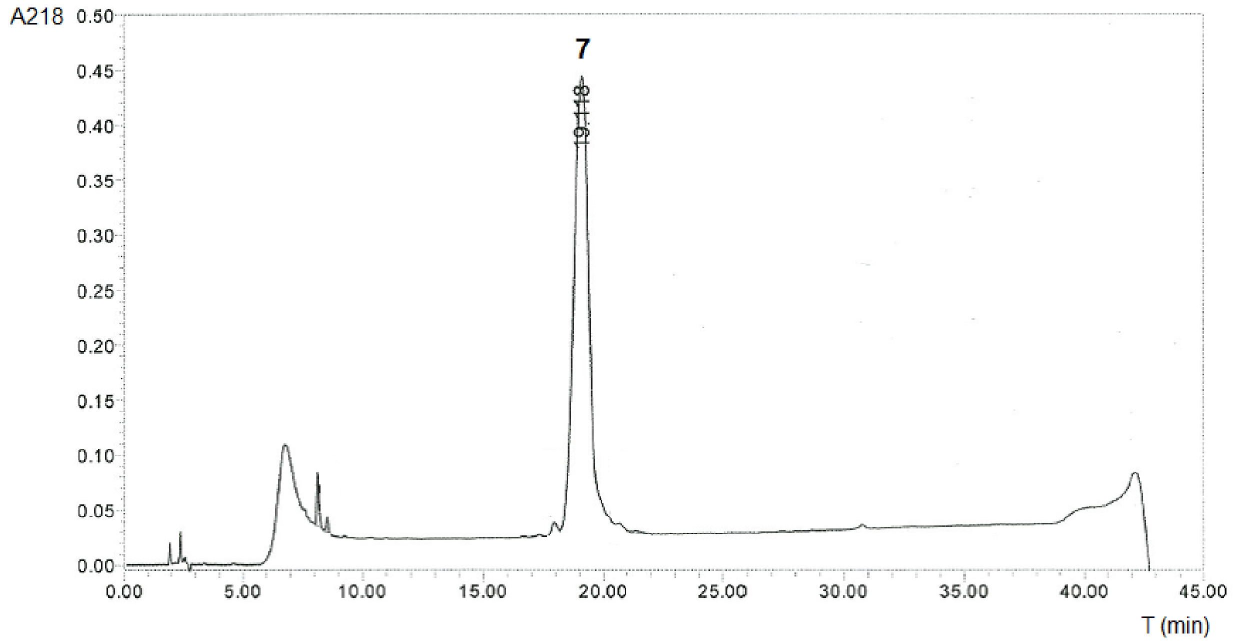

Figure S13. RP-HPLC analysis of analogue 7. The peak of the compound is marked by **7**.

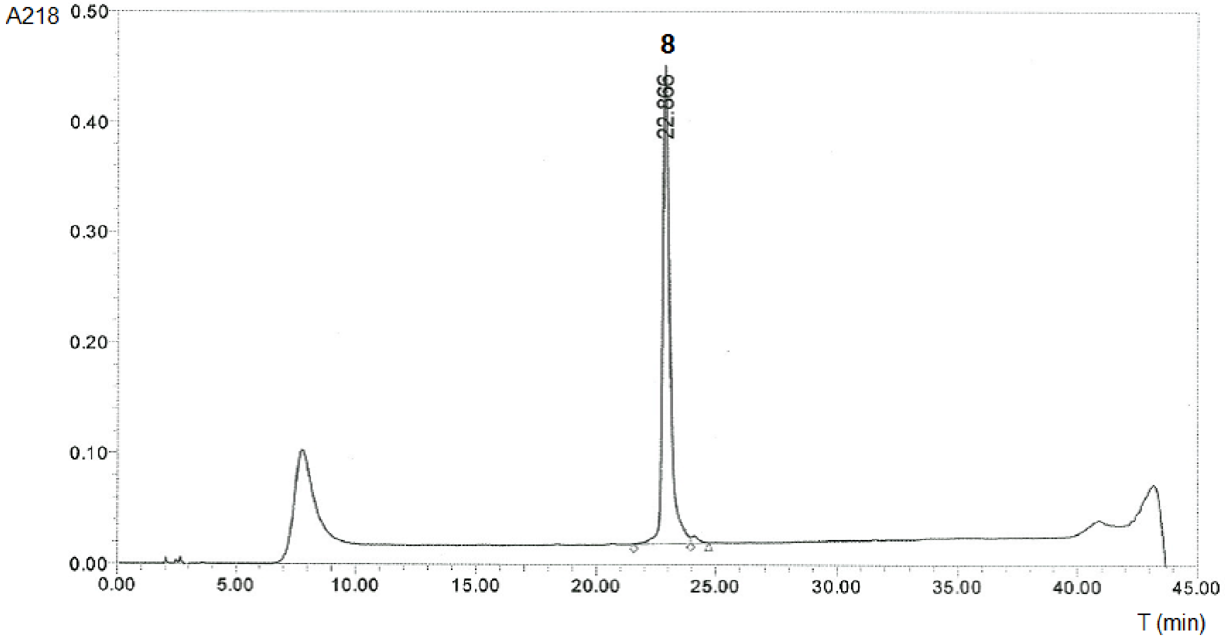

Figure S14. RP-HPLC analysis of analogue 8. The peak of the compound is marked by **8**.

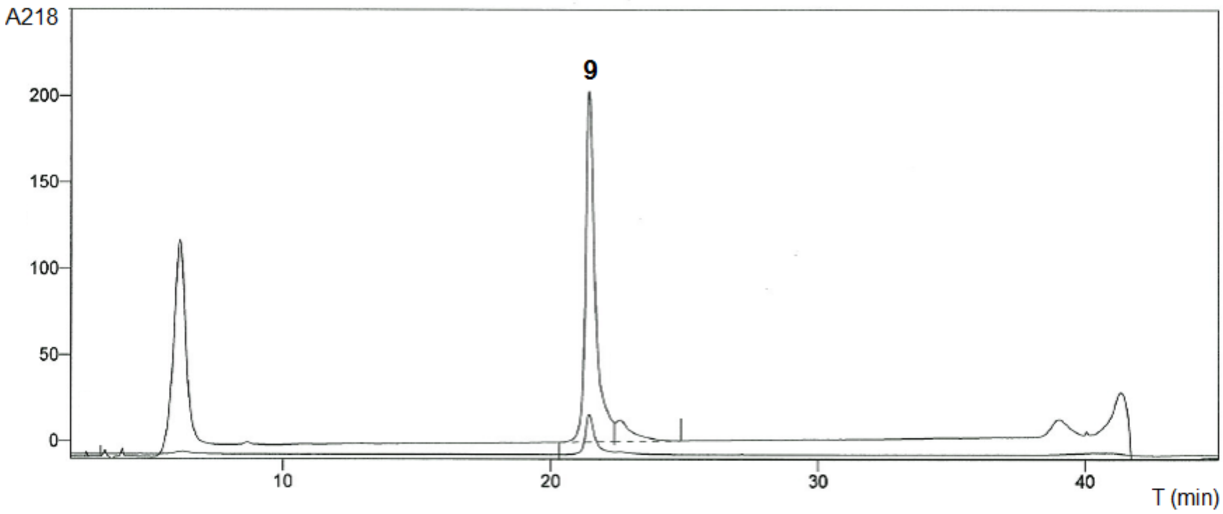

Figure S15. RP-HPLC analysis of analogue 9. The peak of the compound is marked by **9**.

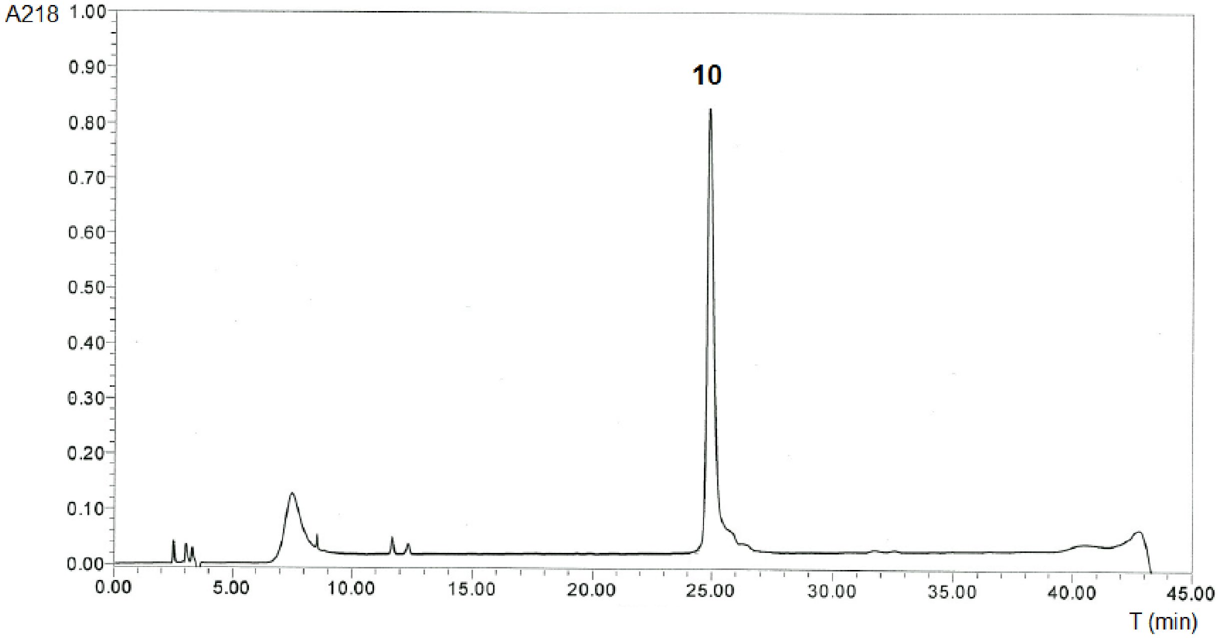

Figure S16. RP-HPLC analysis of analogue 10. The peak of the compound is marked by **10**.

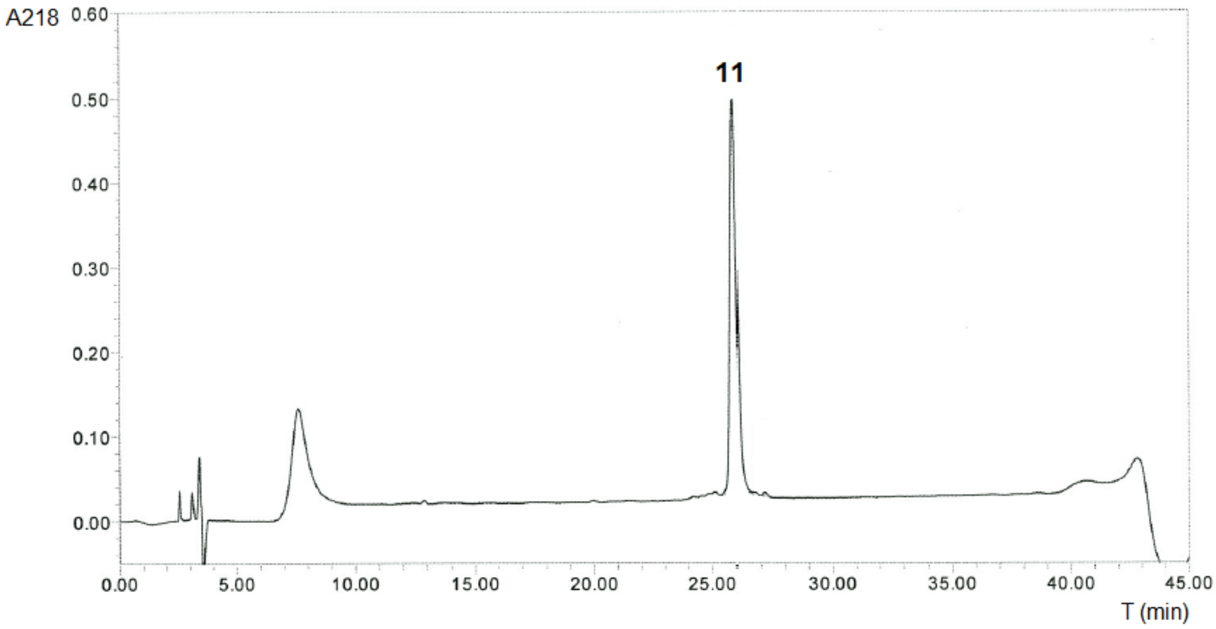

Figure S17. RP-HPLC analysis of analogue 11. The peak of the compound is marked by **11**.

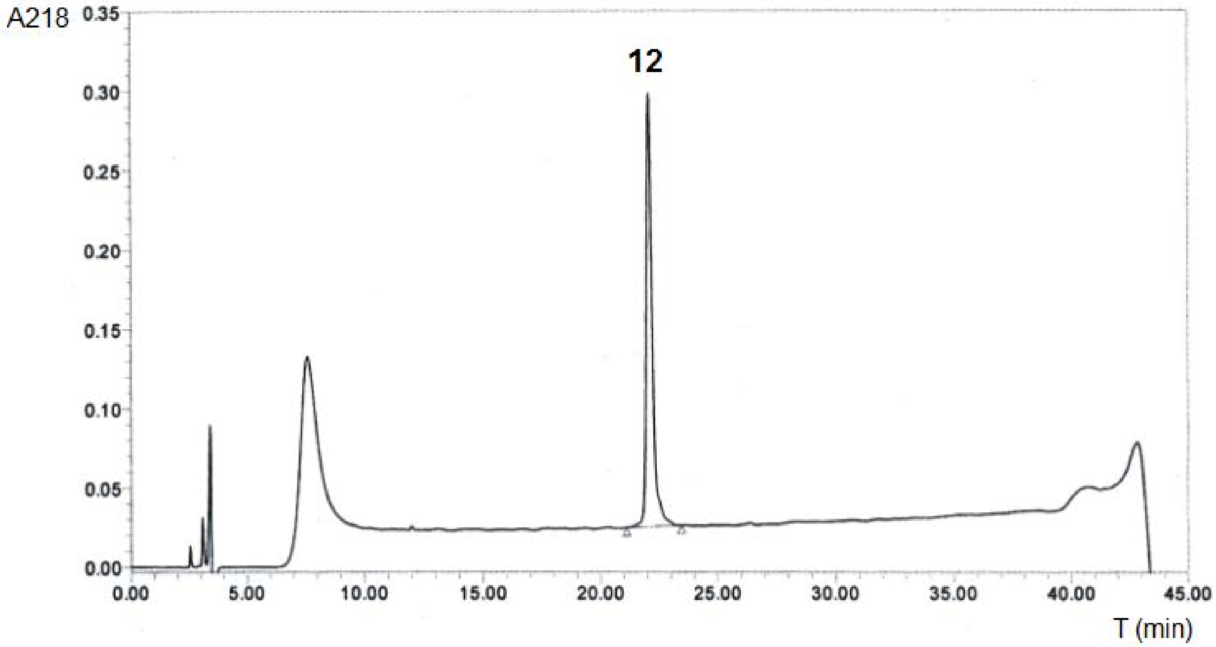

Figure S18. RP-HPLC analysis of analogue 12. The peak of the compound is marked by **12**.

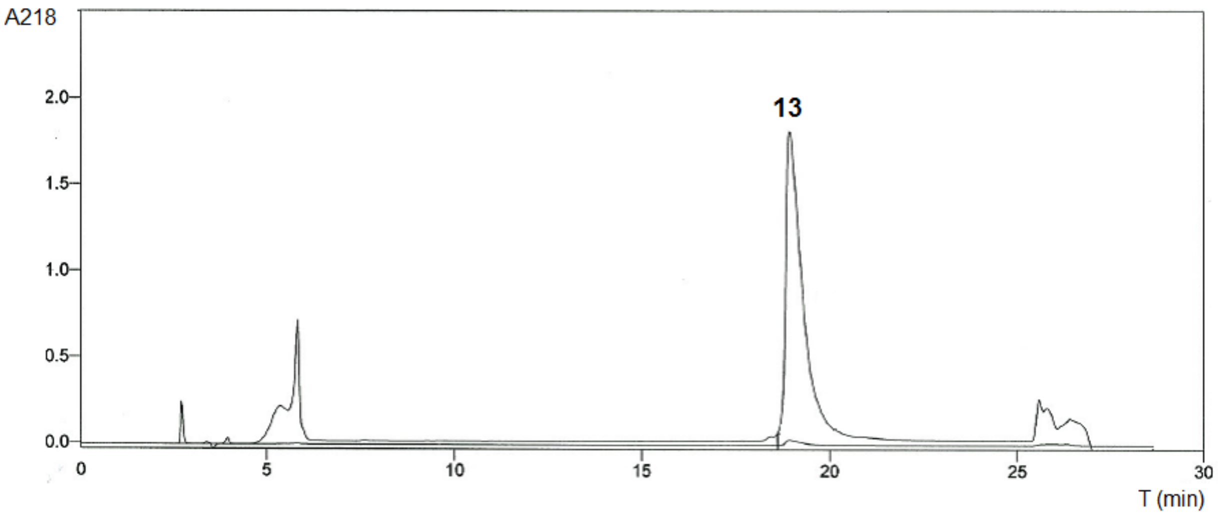

Figure S19. RP-HPLC analysis of analogue 13. The peak of the compound is marked by **13**.

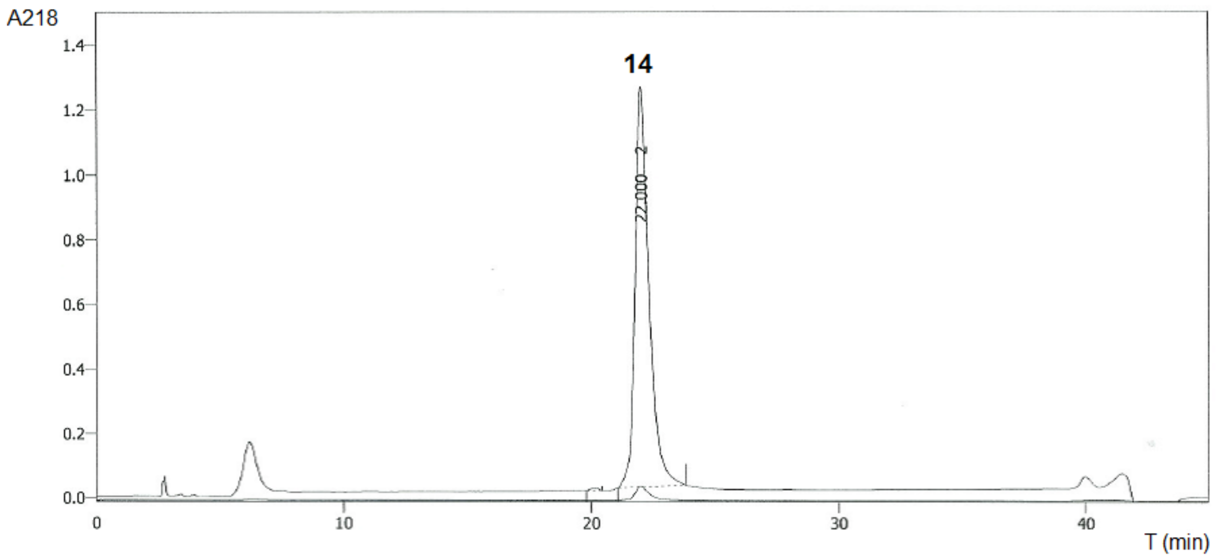

Figure S20. RP-HPLC analysis of analogue 14. The peak of the compound is marked by **14**.

NMR characterization of octapeptides 1a, 6a and 1b-14b.

For the description of NMR data, the residues (positions) in octapeptides are numbered 1-8 starting from the N-terminus. The presence of proline in all studied octapeptides led to the observation of two isomers containing either *trans*- and/or *cis*- X-Pro peptide bond. The *trans*- and *cis*-isomer were evidenced by characteristic NOE contacts around corresponding peptide bond: between αH(X) and δH(Pro) in the *trans*-isomer and/or between αH(X) and αH(Pro) in the *cis*-isomer. In all cases the major isomer is *trans*- and its population appears in the range from >95 % to 61 % (see Supplementary Table 2). The additional tertiary amide bond in compound **14b** (*N*-methyl alanine in position 4) results in the observation of four isomers. Two major isomers contain *trans*- and *cis*- Phe-(*N*Me)Ala peptide bond (in the ratio 72:18, right panel in Supplementary Figure S21) and *trans*- X-Pro bond while two minor isomers have both *cis*- X-Pro peptide bond (left panel in Supplementary Figure S21).

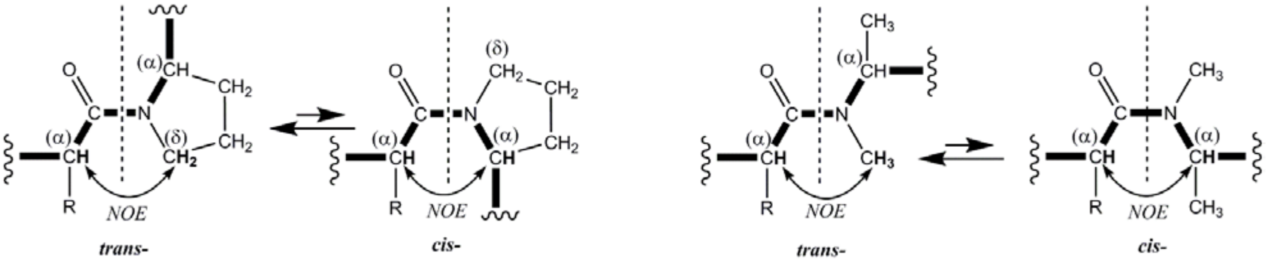

Supplementary Figure S21. N-Methylated peptide bond izomerization. *Trans* to *cis* isomerisation of X-Pro peptide bond (left) and *trans* to *cis* isomerization of Phe-(*N*Me)Ala peptide bond (right).

The temperature coefficients of the amide NH protons are rather high for the most of residues in studied peptides **1a**, **6a** and **1b – 14b** (see Supplementary Table S2). The absolute values below 2.2 ppb that could indicate hydrogen bonded NH proton appear only for NH of Thr-5 in peptides **1b**, **3b** and **4b**, the NH of Tyr-4 in peptide **11b** and the NH of Prg-4 in **9b**. The inspection of models shows that such H-bonds could stabilize a cyclic part of given peptides, *e.g.* (Thr-5)N-H…O=C(Phe-3) in **1b**, **3b** and **4b**.

The complete structure assignment of ^{1}H and ^{13}C signals of major *trans*- isomers in studied peptides **1a**, **6a** and **1b – 14b** is summarized in Supplementary Tables S2-S7.

Supplementary Table S2. **The *trans-/cis-* isomerism in peptide precursors.** The observed ratio of *trans-/cis-* isomers and temperature dependence of amide NH protons of peptides **1a – 14b** in H_2O:D_2O (9:1). n.d. = not determined.

Peptide	X-5/Pro-6		2	3	4	5	7	8
**1a**			**D-Prg**	**Phe**	**Tyr**	**Thr**	**Nle(εN$_3$)**	**Thr**
	trans-<br>*cis-*	90 %<br>10 %	-6.0<br>-6.4	-8.3<br>-6.2	-6.7<br>-5.1	-6.9<br>-7.0	-8.1<br>-8.9	-7.6<br>-6.2
**1b**			**D-Prg**	**Phe**	**Tyr**	**Thr**	**Nle(εN$_3$)**	**Thr**
	trans-<br>*cis-*	81 %<br>19 %	-6.0<br>-3.8	-8.1<br>-9.7	-6.3<br>-4.5	-0.8<br>-7.2	-8.3<br>-6.3	-7.8<br>-5.6
**2b**			**Prg**	**Phe**	**Tyr**	**Thr**	**Nle(εN$_3$)**	**Thr**
	trans-<br>*cis-*	62 %<br>38 %	-6.0<br>-8.1	-8.4<br>-6.2	-3.4<br>-4.1	-6.2<br>-5.5	-4.1<br>-7.1	-6.6<br>-6.6
**3b**			**D-Nle(εN$_3$)**	**Phe**	**Tyr**	**Thr**	**Prg**	**Thr**
	trans-<br>*cis-*	85 %<br>15 %	-9.2<br>-4.8	-8.1<br>-6.2	-5.4<br>-4.2	+0.1<br>-6.8	-5.2<br>-7.8	-9.4<br>-5.9
**4b**			**D-Prg**	**Phe**	**Tyr**	**Thr**	**D-Nle(εN$_3$)**	**Thr**
	trans-<br>*cis-*	73 %<br>27 %	-10.7<br>-8.5	-7.7<br>-9.4	-10.8<br>n.d.	-2.1<br>-2.0	-6.3<br>-4.7	-6.3<br>n.d.
**5b**			**D-Nle(εN$_3$)**	**Phe**	**Tyr**	**Thr**	**D-Prg**	**Thr**
	trans-<br>*cis-*	86 %<br>14 %	-6.4<br>n.d.	-8.9<br>n.d.	-9.7<br>n.d.	-5.9<br>n.d.	-6.9<br>n.d.	-3.5<br>n.d.
**6a**			**Phe**	**Prg**	**Tyr**	**Thr**	**Nle(εN$_3$)**	**Thr**
	trans-<br>*cis-*	92 %<br>8 %	-5.9<br>n.d.	-7.3<br>n.d.	-8.2<br>n.d.	-6.8<br>n.d.	-8.8<br>n.d.	-8.1<br>n.d.
**6b**			**Phe**	**Prg**	**Tyr**	**Thr**	**Nle(εN$_3$)**	**Thr**
	trans-<br>*cis-*	66 %<br>34 %	-7.1<br>-6.7	-5.7<br>-5.4	-5.7<br>-5.9	-3.8<br>-5.5	-5.9<br>-5.7	-9.1<br>-8.1
**7b**			**Phe**	**D-Prg**	**Tyr**	**Thr**	**Nle(εN$_3$)**	**Thr**
	trans-<br>*cis-*	67 %<br>33 %	-6.8<br>-9.2	-9.1<br>-11.2	-7.0<br>-5.5	-3.9<br>-3.8	-6.3<br>-10.2	-9.8<br>-9.1
**8b**			**Phe**	**Phe**	**Nva(δN$_3$)**	**Thr**	**Prg**	**Thr**
	trans-<br>*cis-*	>95%<br>< 5 %	-6.3<br>n.d.	-7.4<br>n.d.	-6.8<br>n.d.	-7.8<br>n.d.	-9.6<br>n.d.	-8.5<br>n.d.
**9b**			**Phe**	**Phe**	**Prg**	**Thr**	**Nva(δN$_3$)**	**Thr**
	trans-<br>*cis-*	61 %<br>39 %	-7.2<br>-7.2	-8.8<br>-8.2	-2.0<br>-7.0	-7.4<br>-5.0	-8.6<br>-11.4	-8.8<br>-8.1
**10b**			**Phe**	**Phe**	**Tyr**	**Nva(δN$_3$)**	**Prg**	**Thr**
	trans-<br>*cis-*	83 %<br>17 %	-7.2<br>n.d.	-8.1<br>n.d.	-8.4<br>n.d.	-7.2<br>n.d.	-9.6<br>n.d.	-7.6<br>n.d.
**11b**			**Phe**	**Phe**	**Tyr**	**Nva(δN$_3$)**	**Gly**	**Prg**
	trans-<br>*cis-*	>95%<br>< 5 %	-6.2<br>n.d.	-7.0<br>n.d.	-1.0<br>n.d.	-6.9<br>n.d.	-7.9<br>n.d.	-7.1<br>n.d.
**12b**			**Gly**	**Phe**	**Tyr**	**Nva(δN$_3$)**	**Prg**	**Thr**
	trans-<br>*cis-*	>95%<br>< 5 %	-5.9<br>n.d.	-7.6<br>n.d.	-7.4<br>n.d.	-7.0<br>n.d.	-8.5<br>n.d.	-6.8<br>n.d.
**13b**			**Gly**	**Phe**	**Nva(δN$_3$)**	**Thr**	**Prg**	**Thr**
	trans-<br>*cis-*	>95%<br>< 5 %	-5.8<br>n.d.	-7.9<br>n.d.	-6.8<br>n.d.	-8.2<br>n.d.	-9.4<br>n.d.	-8.3<br>n.d.
**14b**			**Gly**	**Phe**	**Ala(NMe)**	**Nva(δN$_3$)**	**Prg**	**Thr**
	trans-<br>*cis-*	72 %<br>28 %	-6.5<br>-7.0	-9.8<br>-11.3	--<br>--	-10.8<br>-8.0	-10.5<br>-10.5	-8.3<br>-8.6

Supplementary Table S3. **Proton NMR data of peptides 1a – 4b in H_2O:D_2O (9:1).**

Residue (Proton)	1a	1b	2b	3b	4b
**1**	**Gly**	**Gly**	**Gly**	**Gly**	**Gly**
**$C^{\alpha}H$**	3.87; 3.835	3.73	3.770; 3.692	3.71; 3.65	3.815; 3.748
**2**	**D-Prg**	**D-Prg**	**Prg**	**D-Nle(εN$_3$)**	**D-Prg**
**NH (J(Nα)**	8.670 (6.9)	8.462 (6.6)	8.160 (7.5)	8.601 (6.4)	8.304 (7.1)
**$C^{\alpha}H$**	4.48	4.735	4.727	4.42	4.699
**$C^{\beta}H$**	2.52; 2.47	3.22; 2.90	3.251; 3.008	1.87; 1.54	3.081; 2.908
**$C^{\gamma}H$**	--	--	--	1.92; 1.76	--
**$C^{\delta}H$**	2.315	7.04	7.254	1.15; 0.70	7.32
**$C^{\varepsilon}H$**	--	--	--	4.425; 4.34	--
**3**	**Phe**	**Phe**	**Phe**	**Phe**	**Phe**
**NH (J(Nα)**	8.451 (7.7)	8.636 (6.0)	8.745 (8.4)	8.601 (5.4)	8.378 (6.7)
**$C^{\alpha}H$**	4.67	4.395	4.626	4.15	4.506
**$C^{\beta}H$**	3.13; 2.92	3.036; 2.83	3.132; 2.879	2.92; 2.82	3.098; 2.908
**C_6H_5**	7.21; 7.35; 7.30	7.13; 7.34; 7.28	7.19; 7.34; 7.30	7.32 – 7.26	7.18; 7.35; 7.31
**4**	**Tyr**	**Tyr**	**Tyr**	**Tyr**	**Tyr**
**NH (J(Nα)**	8.280 (7.3)	8.008 (7.7)	7.500 (7.5)	8.273 (7.8)	8.081 (7.9)
**$C^{\alpha}H$**	4.61	4.48	4.671	4.43	4.475
**$C^{\beta}H$**	2.975; 2.94	3.22; 3.05	3.056	3.24; 2.96	3.085
**C_6H_4**	7.10; 6.80	7.16; 6.875	7.050; 6.767	7.18; 6.887	7.114; 6.84
**5**	**Thr**	**Thr**	**Thr**	**Thr**	**Thr**
**NH (J(Nα)**	8.032 (8.2)	7.412 (8.6)	7.889 (8.4)	7.447 (8.4)	7.525 (8.0)
**$C^{\alpha}H$**	4.505	4.725	4.441	4.675	4.628
**$C^{\beta}H$**	4.03	4.08	4.111	4.043	4.272
**$C^{\gamma}H$**	1.17	1.12	1.133	1.211	1.110
**6**	**Pro**	**Pro**	**Pro**	**Pro**	**Pro**
**$C^{\alpha}H$**	4.345	4.31	4.845	4.33	4.374
**$C^{\beta}H$**	2.31; 1.94	2.29; 1.84	2.395; 2.184	2.307; 1.88	2.264; 1.933
**$C^{\gamma}H$**	2.02; 1.97	2.04; 1.94	1.976; 1.847	2.00; 1.95	1.976; 1.927
**$C^{\delta}H$**	3.67; 3.61	3.78; 3.62	3.671; 3.528	3.735; 3.669	3.741; 3.572
**7**	**Nle(εN$_3$)**	**Nle(εN$_3$)**	**Nle(εN$_3$)**	**Prg**	**D-Nle(εN$_3$)**
**NH (J(Nα)**	8.378 (6.8)	8.543 (6.9)	8.642 (7.2)	8.631 (6.7)	7.690 (7.1)
**$C^{\alpha}H$**	4.34	4.27	4.425	4.76	4.401
**$C^{\beta}H$**	1.87; 1.77	1.92; 1.72	1.914; 1.784	3.33; 3.11	1.858
**$C^{\gamma}H$**	1.50; 1.45	1.42; 1.18	1.46	--	1.808; 1.715
**$C^{\delta}H$**	1.62	1.94; 1.86	1.87; 1.82	7.914	1.245; 1.161
**$C^{\varepsilon}H$**	3.33	4.385; 4.29	4.36; 4.27	--	4.346
**8**	**Thr**	**Thr**	**Thr**	**Thr**	**Thr**
**NH (J(Nα)**	8.046 (8.5)	8.065 (8.8)	7.951 (8.5)	8.407 (8.7)	7.959 (8.8)
**$C^{\alpha}H$**	4.365	4.44	4.325	4.51	4.459
**$C^{\beta}H$**	4.34	4.345	4.321	4.44	4.40
**$C^{\gamma}H$**	1.19	1.16	1.157	1.205	1.161

Supplementary Table S4. Proton NMR data of peptides 5b – 8b in H_2O:D_2O (9:1).

Residue (Proton)	5b	6a	6b	7b	8b
**1**	**Gly**	**Gly**	**Gly**	**Gly**	**Gly**
**C$^{\alpha}$H**	3.814; 3.748	3.81; 3.735	3.845; 3.794	3.830; 3.785	3.792; 3.743
**2**	**D-Nle(εN$_3$)**	**Phe**	**Phe**	**Phe**	**Phe**
**NH (J(Nα)**	8.402 (6.7)	8.570 (7.2)	8.660 (6.4)	8.661 (6.2)	8.511 (7.1)
**C$^{\alpha}$H**	4.268	4.665	4.506	4.599	4.645
**C$^{\beta}$H**	1.57; 1.509	3.02; 2.95	2.948; 2.82	3.074; 3.004	3.018; 2.975
**C_6H_5**	--	7.21; 7.33; 7.30	7.30 – 7.25; 7.01	7.28; 7.40; 7.34	7.20; 7.34; 7.31
**C$^{\gamma}$H**	1.841	--	--	--	--
**C$^{\delta}$H**	1.225; 1.066	--	--	--	--
**C$^{\epsilon}$H**	4.417; 4.358	--	--	--	--
**3**	**Phe**	**Prg**	**Prg**	**D-Prg**	**Phe**
**NH (J(Nα)**	8.302 (7.5)	8.381 (7.6)	8.199 (7.8)	8.223 (8.2)	8.221 (7.6)
**C$^{\alpha}$H**	4.468	4.495	4.578	4.646	4.558
**C$^{\beta}$H**	3.055; 2.942	2.64; 2.61	3.077; 2.97	3.288; 2.741	2.987
**C_6H_5**	7.11; 7.33; 7.30	--	--	--	7.20; 7.34; 7.31
**C$^{\delta}$H**	--	2.42	7.538	7.385	--
**4**	**Tyr**	**Tyr**	**Tyr**	**Tyr**	**Nva(δN$_3$)**
**NH (J(Nα)**	8.138 (6.8)	8.262 (7.2)	8.003 (6.2)	7.233 (6.7)	7.984 (6.7)
**C$^{\alpha}$H**	4.454	4.595	4.344	4.538	4.396
**C$^{\beta}$H**	3.164; 3.048	2.98	2.941; 2.809	2.963; 2.924	1.874; 1.754
**C_6H_4**	7.163; 6.862	7.13; 6.81	7.16; 6.82	7.025; 6.801	2.117; 1.874
**C$^{\gamma}$H**	--	--	--	--	4.48; 4.33
**C$^{\delta}$H**	--	--	--	--	--
**5**	**Thr**	**Thr**	**Thr**	**Thr**	**Thr**
**NH (J(Nα)**	7.669 (8.0)	8.034 (8.2)	7.760 (9.4)	7.836 (9.1)	8.081 (6.6)
**C$^{\alpha}$H**	4.635	4.53	4.409	4.423	4.317
**C$^{\beta}$H**	4.242	4.04	4.111	4.111	4.344
**C$^{\gamma}$H**	1.132	1.18	1.181	1.207	1.302
**6**	**Pro**	**Pro**	**Pro**	**Pro**	**Pro**
**C$^{\alpha}$H**	4.418	4.35	4.647	4.680	4.096
**C$^{\beta}$H**	2.234; 1.975	2.30; 1.97	2.464; 2.122	2.447; 2.145	2.276; 1.806
**C$^{\gamma}$H**	2.004; 1.981	2.01; 1.95	2.426; 1.932	1.947	2.143; 1.983
**C$^{\delta}$H**	3.732; 3.647	3.66; 3.62	3.61; 3.495	3.626; 3.509	3.803; 3.576
**7**	**D-Prg**	**Nle(εN$_3$)**	**Nle(εN$_3$)**	**Nle(εN$_3$)**	**Prg**
**NH (J(Nα)**	7.895 (7.8)	8.365 (7.0)	8.822 (6.0)	8.798 (6.0)	8.717 (5.9)
**C$^{\alpha}$H**	4.342	4.345	4.331	4.289	4.627
**C$^{\beta}$H**	3.317; 3.283	1.87; 1.77	1.877; 1.813	1.988; 1.852	3.380; 3.106
**C$^{\gamma}$H**	--	1.48	1.625; 1.534	1.602; 1.492	--
**C$^{\delta}$H**	7.883	1.63	2.122; 1.933	2.076; 1.898	???
**C$^{\epsilon}$H**	--	3.335	4.46; 4.19	4.462; 4.187	--
**8**	**Thr**	**Thr**	**Thr**	**Thr**	**Thr**
**NH (J(Nα)**	7.890 (7.6)	8.067 (8.6)	8.279 (8.7)	8.030 (8.8)	8.246 (8.5)
**C$^{\alpha}$H**	4.769	4.39	4.466	4.491	4.522
**C$^{\beta}$H**	4.322	4.35	4.398	4.410	4.436
**C$^{\gamma}$H**	1.176	1.18	1.190	1.201	1.196

Supplementary Table S5. Proton NMR data of peptides 9b – 14b in H_2O:D_2O (9:1).

Residue (Proton)	9b	10b	11b	12b	13b	14b
**1**	**Gly**	**Gly**	**Gly**	**Gly**	**Gly**	**Gly**
**C$^{\alpha}$H**	3.731; 3.654	3.78; 3.72	3.77; 3.72	3.87	3.868	3.87
**2**	**Phe**	**Phe**	**Phe**	**Gly**	**Gly**	**Gly**
**NH (J(Nα)**	8.436 (7.7)	8.487 (7.2)	8.471 (7.1)	8.472 (5.8)	8.487 (5.9)	8.475 (6.0)
**C$^{\alpha}$H**	4.684	4.62	4.61	3.96	3.974	3.97
**C$^{\beta}$H**	3.034; 2.851	2.97; 2.92	2.97; 2.92	--	--	--
**C_6H_5**	7.21; 7.34; 7.30	7.17; 7.33; 7.31	7.16; 7.31; 7.31	--	--	--
**3**	**Phe**	**Phe**	**Phe**	**Phe**	**Phe**	**Phe**
**NH (J(Nα)**	8.544 (7.8)	8.210 (7.6)	8.192 (7.6)	8.207 (7.1)	8.251 (7.0)	8.350 (7.0)
**C$^{\alpha}$H**	4.686	4.57	4.54	4.61	4.618	5.045
**C$^{\beta}$H**	3.180; 3.002	2.97 (2H)	2.96 (2H)	3.03; 3.00	3.066; 3.030	3.07; 3.01
**C_6H_5**	7.21; 7.34; 7.30	7.19; 7.33; 7.31	7.18; 7.31; 7.31	7.21; 7.35; 7.31	7.23; 7.35; 7.30	7.40 – 7.24
**4**	**Prg**	**Tyr**	**Tyr**	**Tyr**	**Nva(δN_3)**	**Ala(*N*Me)**
**NH (J(Nα)**	7.660 (7.2)	8.037 (7.2)	8.001 (7.4)	8.137 (7.1)	8.084 (6.8)	--
**C$^{\alpha}$H**	4.720	4.37	4.31	4.42	4.455	4.69
**C$^{\beta}$H**	3.173	2.87; 2.76	2.87; 2.78	2.91; 2.77	1.88	1.24
**C_6H_4**	--	7.02; 6.76	7.04; 6.78	7.01; 6.75	--	--
**C$^{\gamma}$H**	--	--	--	--	2.12; 1.78	--
**C$^{\delta}$H**	7.783	--	--	--	4.29	--
**N-Me**	--	--	--	--	--	2.89
**5**	**Thr**	**Nva(δN_3)**	**Nva(δN_3)**	**Nva(δN_3)**	**Thr**	**Nva(δN_3)**
**NH (J(Nα)**	8.220 (8.2)	7.840 (7.2)	7.909 (7.5)	7.840 (7.3)	8.109 (6.5)	7.765 (5.4)
**C$^{\alpha}$H**	4.359	4.26	4.385	4.285	4.328	4.32
**C$^{\beta}$H**	3.984	1.68; 1.47	1.56; 1.06	1.71; 1.49	4.343	1.66; 1.43
**C$^{\gamma}$H**	1.147	2.19; 1.32	2.06; 1.81	2.23; 1.32	1.304	2.27; 1.64
**C$^{\delta}$H**	--	4.40; 4.29	4.45; 4.33	4.44; 4.32	--	4.46; 4.33
**6**	**Pro**	**Pro**	**Pro**	**Pro**	**Pro**	**Pro**
**C$^{\alpha}$H**	4.868	4.29	4.285	4.25	4.102	4.46
**C$^{\beta}$H**	2.363; 2.098	2.02 (2H)	2.29; 1.92	2.06; 2.01	2.278; 1.811	2.06; 2.01
**C$^{\gamma}$H**	1.972; 1.862	2.11; 1.95	2.05; 1.97	2.12; 1.95	2.141; 1.99	2.17; 2.03
**C$^{\delta}$H**	3.665; 3.519	3.50; 3.43	3.64; 3.55	3.51; 3.40	3.813; 3.581	3.79; 3.61
**7**	**Nva(δN_3)**	**Prg**	**Gly**	**Prg**	**Prg**	**Prg**
**NH (J(Nα)**	8.626 (8.2)	8.216 (8.9)	8.514 (6.3; 5.9)	8.217 (8.7)	8.706 (6.9)	8.254 (8.9)
**C$^{\alpha}$H**	4.578	5.05	4.18; 3.73	5.105	4.613	5.11
**C$^{\beta}$H**	1.77; 1.096	3.37; 3.12	--	3.385; 3.15	3.363; 3.119	3.39; 3.15
**C$^{\gamma}$H**	1.772; 1.647	--	--	--	--	--
**C$^{\delta}$H**	4.514	7.64	--	7.74	8.083	7.70
**8**	**Thr**	**Thr**	**Prg**	**Thr**	**Thr**	**Thr**
**NH (J(Nα)**	8.428 (8.5)	8.277 (8.5)	7.835 (7.0)	8.217 (8.7)	8.227 (8.7)	8.320 (8.4)
**C$^{\alpha}$H**	4.682	4.48	4.59	4.55	4.494	4.51
**C$^{\beta}$H**	4.408	4.42	3.42; 3.37	4.45	4.42	4.43
**C$^{\gamma}$H**	1.200	1.22	--	1.23	1.190	1.22
**C$^{\delta}$H**	--	--	7.76	--	--	--

Supplementary Table 6. Carbon-13 NMR data of peptides 1a – 6b in H_2O:D_2O (9:1).

Residue (Carbon)	1a	1b	2b	3b	4b	5b	6a	6b
**1**	**Gly**	**Gly**	**Gly**	**Gly**	**Gly**	**Gly**	**Gly**	**Gly**
**CO**	169.86	169.37	169.13	169.28	169.49	169.82	169.59	169.57
**C$^{\alpha}$**	43.26	43.30	43.32	43.26	43.39	43.26	43.19	43.21
**2**	**D-Prg**	**D-Prg**	**Prg**	**D-Nle(εN$_3$)**	**D-Prg**	**D-Nle(εN$_3$)**	**Phe**	**Phe**
**CO**	174.26	174.57	172.14	175.88	174.75	176.10	175.39	174.55
**C$^{\alpha}$**	55.06	55.74	55.28	55.78	56.30	56.27	57.75	58.73
**C$^{\beta}$**	24.00	30.21	30.77	31.82	30.24	32.92	40.11	40.15
**C$^{\gamma}$**	81.41	144.15	143.83	30.35	144.61	31.25	138.47	138.49
**C$^{\delta}$**	75.27	127.30	126.64	21.89	127.34	24.01	131.89	131.86
**C$^{\varepsilon}$**	--	--	--	51.49	--	52.50	131.50	131.42
**C$^{\zeta}$**	--	--	--	--	--	--	129.96	129.81
**3**	**Phe**	**Phe**	**Phe**	**Phe**	**Phe**	**Phe**	**Prg**	**Prg**
**CO**	175.21	175.92	174.33	176.20	175.66	175.78	173.63	173.07
**C$^{\alpha}$**	57.54	59.44	57.99	59.72	58.63	58.50	57.57	58.02
**C$^{\beta}$**	39.82	38.68	40.02	38.75	39.16	39.21	23.99	29.88
**C$^{\gamma}$**	138.88	139.47	139.18	138.73	139.25	139.02	81.90	144.30
**C$^{\delta}$**	131.86	131.77	131.86	131.62	131.85	131.77	75.17	n.d.
**C$^{\varepsilon}$**	131.52	131.62	131.48	131.43	131.65	131.50	--	--
**C$^{\zeta}$**	129.90	130.02	129.87	129.88	130.07	129.87	--	--
**4**	**Tyr**	**Tyr**	**Tyr**	**Tyr**	**Tyr**	**Tyr**	**Tyr**	**Tyr**
**CO**	175.02	175.45	174.18	176.04	175.52	175.92	175.12	174.34
**C$^{\alpha}$**	57.92	58.68	57.38	59.09	49.16	59.29	57.92	58.10
**C$^{\beta}$**	39.12	38.13	39.10	38.29	37.71	38.10	39.08	39.01
**C$^{\gamma}$**	130.52	131.16	131.25	131.58	130.78	130.88	130.39	130.59
**C$^{\delta}$**	133.28	133.23	133.54	133.28	133.26	133.25	133.30	133.48
**C$^{\varepsilon}$**	118.13	118.47	118.32	118.43	118.43	118.46	118.19	118.38
**C$^{\zeta}$**	157.24	157.47	157.55	157.39	157.44	157.41	157.33	157.32
**5**	**Thr**	**Thr**	**Thr**	**Thr**	**Thr**	**Thr**	**Thr**	**Thr**
**CO**	171.92	171.84	172.14	171.44	172.88	173.00	172.01	172.47
**C$^{\alpha}$**	59.15	58.97	58.70	59.28	n.d.	55.72	59.20	60.79
**C$^{\beta}$**	70.18	70.35	70.68	70.66	69.48	69.69	70.13	70.13
**C$^{\gamma}$**	21.71	21.38	21.13	21.43	21.67	21.49	21.22	21.59
**6**	**Pro**	**Pro**	**Pro**	**Pro**	**Pro**	**Pro**	**Pro**	**Pro**
**CO**	176.83	177.20	176.14	176.36	176.85	177.05	176.61	176.50
**C$^{\alpha}$**	62.98	63.40	62.64	62.96	64.03	63.84	62.99	62.29
**C$^{\beta}$**	32.21	32.09	32.04	32.27	31.63	31.86	32.19	34.54
**C$^{\gamma}$**	27.28	27.66	27.48	27.39	27.50	27.27	27.27	25.37
**C$^{\delta}$**	51.03	51.03	50.23	50.94	50.98	50.99	51.02	50.53
**7**	**Nle(εN$_3$)**	**Nle(εN$_3$)**	**Nle(εN$_3$)**	**Prg**	**D-Nle(εN$_3$)**	**D-Prg**	**Nle(εN$_3$)**	**Nle(εN$_3$)**
**CO**	176.64	176.52	175.57	175.82	176.50	174.69	176.89	176.25
**C$^{\alpha}$**	56.69	56.78	56.27	56.53	56.21	62.31	56.67	56.44
**C$^{\beta}$**	33.21	32.73	32.88	29.54	31.19	29.98	33.22	32.75
**C$^{\gamma}$**	25.09	24.70	24.88	145.70	32.88	145.32	25.08	25.08
**C$^{\delta}$**	30.28	30.85	31.30	126.97	24.04	126.96	30.28	30.45
**C$^{\varepsilon}$**	53.60	52.66	52.85	--	52.82	--	53.62	53.41
**8**	**Thr**	**Thr**	**Thr**	**Thr**	**Thr**	**Thr**	**Thr**	**Thr**
**CO**	177.26	177.34	177.82	177.36	176.55	177.65	176.98	177.74
**C$^{\alpha}$**	61.57	60.78	61.88	60.90	60.81	59.46	61.37	60.87
**C$^{\beta}$**	70.33	70.10	70.44	70.04	70.02	70.52	70.25	70.20
**C$^{\gamma}$**	21.17	21.58	21.80	21.63	21.72	21.92	21.87	21.88

Supplementary Table S7. Carbon-13 NMR data of peptides 7b – 14b in $H_2O:D_2O$ (9:1).

Residue (Carbon)	7b	8b	9b	10b	11b	12b	13b	14b
**1**	**Gly**	**Gly**	**Gly**	**Gly**	**Gly**	**Gly**	**Gly**	**Gly**
**CO**	169.89	169.54	169.41	169.50	169.37	170.35	n.d.	170.48
**C^{α}**	43.20	43.22	43.14	43.20	42.93	43.09	43.35	43.37
**2**	**Phe**	**Phe**	**Phe**	**Phe**	**Phe**	**Gly**	**Gly**	**Gly**
**CO**	175.70	175.89	175.42	174.98	174.20	173.33	173.50	173.12
**C^{α}**	58.61	57.91	57.54	57.82	57.78	44.78	44.94	44.86
**C^{β}**	39.72	40.20	40.18	40.10	40.14	--	--	--
**C^{γ}**	138.78	138.77	139.19	138.75	138.74	--	--	--
**C^{δ}**	131.87	131.97	131.95	131.87	131.86	--	--	--
**C^{ε}**	131.63	131.48	131.45	131.38	131.47	--	--	--
**C^{ζ}**	130.12	129.96	129.95	129.93	129.94	--	--	--
**3**	**D-Prg**	**Phe**	**Phe**	**Phe**	**Phe**	**Phe**	**Phe**	**Phe**
**CO**	174.14	174.19	174.80	174.27	174.23	175.06	175.04	175.29
**C^{α}**	55.39	57.52	57.54	57.47	57.34	57.59	57.77	56.85
**C^{β}**	29.20	40.07	39.83	40.12	40.08	39.93	40.01	39.59
**C^{γ}**	144.16	138.75	138.85	138.77	138.72	138.74	138.76	138.59
**C^{δ}**	127.27	131.86	131.92	131.97	131.96	131.91	131.92	132.12
**C^{ε}**	--	131.42	131.44	131.47	131.39	131.43	131.47	131.45
**C^{ζ}**	--	129.84	129.83	129.82	129.94	129.90	129.90	130.01
**4**	**Tyr**	**Nva(δN_3)**	**Prg**	**Tyr**	**Tyr**	**Tyr**	**Nva(δN_3)**	**Ala(NMe)**
**CO**	175.45	175.06	172.63	173.79	173.67	173.78	175.99	175.53
**C^{α}**	57.65	55.41	54.31	57.41	56.20	57.50	55.52	53.37
**C^{β}**	28.90	32.10	30.04	39.17	39.30	39.11	32.01	15.97
**C^{γ}**	131.00	28.04	144.78	130.37	130.44	130.39	28.05	--
**C^{δ}**	133.42	53.04	128.55	133.21	133.26	133.20	52.84	--
**C^{ε}**	118.37	--	--	118.08	117.97	117.94	--	--
**C^{ζ}**	157.36	--	--	157.23	157.12	157.07	--	--
**NMe**	--	--	--	--	--	--	--	34.67
**5**	**Thr**	**Thr**	**Thr**	**Nva(δN_3)**	**Nva(δN_3)**	**Nva(δN_3)**	**Thr**	**Nva(δN_3)**
**CO**	172.42	171.76	172.10	173.24	172.83	172.96	171.77	175.29
**C^{α}**	58.47	61.35	58.84	52.40	52.63	52.21	61.37	54.24
**C^{β}**	70.01	67.56	71.03	29.74	31.30	29.65	67.56	29.65
**C^{γ}**	21.68	22.11	20.37	27.70	27.20	27.63	22.09	27.46
**C^{δ}**	--	--	--	52.38	52.19	52.96	--	52.45
**6**	**Pro**	**Pro**	**Pro**	**Pro**	**Pro**	**Pro**	**Pro**	**Pro**
**CO**	176.30	175.99	175.74	176.35	174.96	176.51	176.05	176.26
**C^{α}**	62.36	64.92	63.39	62.50	63.16	62.55	64.93	62.65
**C^{β}**	34.50	31.63	34.50	30.46	31.98	30.60	31.62	30.75
**C^{γ}**	25.06	28.04	24.64	27.31	27.26	27.33	28.11	27.91
**C^{δ}**	50.52	50.10	49.85	50.44	50.70	50.41	50.11	50.73
**7**	**Nle(εN_3)**	**Prg**	**Nva(δN_3)**	**Prg**	**Gly**	**Prg**	**Prg**	**Prg**
**CO**	176.45	n.d.	175.34	175.23	169.51	175.03	170.53	174.68
**C^{α}**	56.60	56.01	55.34	54.47	46.12	54.14	56.16	54.27
**C^{β}**	32.71	29.46	30.59	30.79	--	30.60	29.44	30.31
**C^{γ}**	25.17	n.d.	28.13	146.58	--	145.85	145.52	n.d.
**C^{δ}**	30.69	n.d.	50.64	125.95	--	126.75	127.48	n.d.
**C^{ε}**	53.23	--	--	--	--	--	--	--
**8**	**Thr**	**Thr**	**Thr**	**Thr**	**Prg**	**Thr**	**Thr**	**Thr**
**CO**	177.62	177.23	176.33	176.82	178.10	176.24	177.23	176.56
**C^{α}**	60.76	61.09	61.18	61.40	57.51	60.86	60.99	61.22
**C^{β}**	70.24	70.06	69.80	69.99	29.56	69.73	70.12	69.93
**C^{γ}**	21.76	21.58	21.63	21.71	133.70	21.56	21.60	21.68
**C^{δ}**	--	--	--	--	125.90	--	--	--

Supplementary Table S8. The numbers of observed NOE peaks, additional constraints and structural statistics for calculated structures of analogue 8.

	Analogue **8*** pH 1.9		Analogue **8*** pH 8	
Non-redundant distance and angle				
Total number of NOE constraints	840		477	
Short-range NOEs				
Intra-residue (i = j)	218		135	
Sequential (| i - j | = 1)	237		114	
Medium-range NOEs (1 < | i - j | < 5)	184		95	
Long-range NOEs (| i - j | ≥ 5)	198		130	
Torsion angles	52		-	
Hydrogen bond restrains	49		30	
Total number of restricting constraints	941			
Total restricting constraints per restrained residue	19.2		11.0	
Residual constraint violations				
Distance violations per structure				
0.1 – 0.2 Å	11.25		9.47	
0.2 – 0.5 Å	4.69		6.33	
> 0.5 Å	0		0	
r.m.s. of distance violation per	0.04 Å		0.06 Å	
Maximum distance violation	0.50 Å		0.50 Å	
Dihedral angle violations per structure				
1 – 10 °	0.97		-	
> 10 °	0		-	
r.m.s. of dihedral violations per	0.48 °		-	
Maximum dihedral angle violation	5.00 °		-	
Ramachandran plot summary from				
Most favoured regions	96.3 %		90.9 %	
Additionally allowed regions	3.7 %		8.7 %	
Generously allowed regions	0.0 %		0.3 %	
Disallowed regions	0.0 %		0.1 %	
r.m.s.d. to the mean structure	*ordered*	*all*	*ordered*	*all*
All backbone atoms	0.9 Å	1.2 Å	0.5 Å	1.0 Å
All heavy atoms	1.2 Å	1.5 Å	0.9 Å	1.5 Å

*Analogue **8**: Cyclo[G^{B23}FF-**Nva(δN_3)**-TP-**Prg**-T^{B30}]-insulin

Supplementary Table S9. The numbers of observed NOE peaks, additional constraints and structural statistics for calculated structures of analogue 12.

	Analogue **12*** pH 1.9	
Non-redundant distance and angle		
Total number of NOE constraints	894	
Short-range NOEs		
Intra-residue (i = j)	227	
Sequential (| i - j | = 1)	225	
Medium-range NOEs (1 < | i - j | < 5)	218	
Long-range NOEs (| i - j | ≥ 5)	221	
Torsion angles	-	
Hydrogen bond restrains	64	
Total number of restricting constraints		
Total restricting constraints per restrained residue	19.6	
Residual constraint violations		
Distance violations per structure		
0.1 – 0.2 Å	7.17	
0.2 – 0.5 Å	3.03	
> 0.5 Å	0	
r.m.s. of distance violation per	0.03 Å	
Maximum distance violation	0.50 Å	
Dihedral angle violations per structure		
1 – 10 °	-	
> 10 °	-	
r.m.s. of dihedral violations per	-	
Maximum dihedral angle violation	-	
Ramachandran plot summary from		
Most favoured regions	88.7 %	
Additionally allowed regions	11.1 %	
Generously allowed regions	0.3 %	
Disallowed regions	0.0 %	
r.m.s.d. to the mean structure	*ordered*	*all residues*
All backbone atoms	0.3 Å	0.5 Å
All heavy atoms	0.7 Å	0.8 Å

* Analogue **12**: Cyclo[G^{B23}-**G**-FY-**Nva(δN_3)**-P-**Prg**-T^{B30}]-insulin

Supplementary Table 10. Data collection and refinement statistics for analogues 2, 10 and 11.

	Analogue **2***	Analogue **10***	Analogue **11****
Space group	$P2_12_12$	$C2$	$P3_1$
Cell dimensions			
a, *b*, *c* (Å)	55.44 56.86 60.00	66.10 45.97 43.93	60.99 60.99 81.92
α, β, γ (β)	90.0 90.0 90.0	90.0 128.5 90.0	90.0 90.0 120.0
Resolution (Å)	41.28 – 1.70(1.73-1.70)[§]	34.38 – 1.90(1.94-1.90)	50.0 – 1.54(1.57-1.54)
R_{sym}	0.070(0.253)	0.059(0.42)	0.068(0.488)
$<I / \sigma(I)>$	15.7(4.3)	12.7(3.0)	15.2(3.5)
Completeness (%)	92.6(60.5)	97.7(92.7)	100.0(100.0)
**Refinement**			
Resolution (Å)	60.01 - 1.70	34.38 – 1.90	50.0 – 1.54
No. reflections	18898	7579	13606
R_{work} / R_{free}	0.196/0.232	0.235/0.304	0.161/0.195
No. atoms	1606	899	2913
Protein	1391	805	2464
Ligand/ion	15	-	70/4
Water	200	94	375
B-factors			
Protein	19.46	24.61	18.55
Ligand/ion	37.71	-	14.65/10.0
Water	30.21	26.10	31.19
R.m.s. deviations			
Bond lengths (Å)	0.019	0.020	0.025
Bond angles (°)	2.36	2.30	2.23

Analogue **2**: Cyclo[G^{B23}-**Prg**-FYTP-**Nle(εN$_3$)**-T^{B30}]-insulin
Analogue **10**: Cyclo[G^{B23}FFY-**Nva(δN$_3$)**-P-**Prg**-T^{B30}]-insulin
Analogue **11**: Cyclo[G^{B23}FFY-**Nva(δN$_3$)**-P**G**-**Prg**^{B30}]-insulin

*DLS – Diamond Light Source, Didcot, UK, **ESRF – European Synchrotron Radiation Facility, Grenoble, France; [§]Values in parentheses are for highest-resolution shell; All X-ray data were collected on one crystal only.
